# Inactivity-induced NR4A3 downregulation in human skeletal muscle affects glucose metabolism and translation: Insights from *in vitro* analysis

**DOI:** 10.1016/j.molmet.2025.102200

**Published:** 2025-07-01

**Authors:** Jonathon A.B. Smith, Brendan M. Gabriel, Aidan J. Brady, Ahmed M. Abdelmoez, Mladen Savikj, Shane C. Wright, Stefania Koutsilieri, Romain Barrès, Volker M. Lauschke, Anna Krook, Juleen R. Zierath, Nicolas J. Pillon

**Affiliations:** 1 Karolinska Institutet, Department of Physiology and Pharmacology, Stockholm, Sweden; 2 Karolinska Institutet, Department of Molecular Medicine and Surgery, Stockholm, Sweden; 3 Novo Nordisk Foundation Center for Basic Metabolic Research, Faculty of Health and Medical Sciences, University of Copenhagen, Copenhagen, Denmark

**Keywords:** Skeletal muscle, Inactivity, Metabolism, Mitochondria, Protein synthesis

## Abstract

**Objective:**

Physical activity promotes health, whereas inactivity is associated with metabolic impairment. The transcription factor nuclear receptor subfamily 4 group A member 3 (NR4A3) is a pleiotropic regulator of skeletal muscle exercise adaptation and metabolism. However, the consequence of lower NR4A3 expression remains largely unexplored. We investigated the impact of NR4A3 downregulation on human skeletal muscle metabolism.

**Methods:**

Published transcriptomic datasets from human bed rest and limb immobilisation studies were curated to meta-analyse the effect of physical inactivity on skeletal muscle *NR4A3* levels. In primary human skeletal myotubes, siRNA and lentivirus were used to silence and overexpress *NR4A3*, respectively. Basal and stimulated (insulin ± leucine) signal transduction was determined by immunoblot analysis. Effects on glucose, fatty acid, and protein metabolism were measured using radiolabelled substrate assays. Lactate production was assessed in culture supernatant by colourimetry. Cell morphology was analysed by immunocytochemistry and gene expression was quantified by RT-qPCR.

**Results:**

Physical inactivity decreased skeletal muscle *NR4A3* (−27%), concomitant with pathways related to mitochondrial function, cytoskeleton organization, chromatin regulation, protein synthesis and degradation. Silencing of *NR4A3* reduced glucose oxidation (−18%) and increased lactate production (+23%) *in vitro*. This coincided with greater signalling downstream of AMPK and elevated rates of basal (+26%) and FCCP-stimulated (+55%) fatty acid oxidation. NR4A3 downregulation lowered protein synthesis (−25%), and impaired mTORC1 signalling and ribosomal transcription. Alternatively, overexpression of the canonical NR4A3 protein isoform (+290%) augmented translation and total cellular protein content, which protected myotubes against dexamethasone-induced atrophy. Moreover, partial restoration of *NR4A3* levels rescued glucose oxidation in *NR4A3*-silenced muscle cells and restored phosphorylation of mTORC1 substrates. NR4A3 depletion reduced myotube area (−48%) and further altered protein and gene expression of key contractile elements in skeletal muscle.

**Conclusions:**

Our study connects reduced NR4A3 expression with physical inactivity and indicates that NR4A3 downregulation in human skeletal muscle has adverse effects on glucose metabolism and protein synthesis. Thus, decrements in NR4A3 abundance could be causal in the deleterious health consequences resulting from sedentary lifestyles and targeting NR4A3 may offer new avenues for combating conditions such as disuse muscle atrophy.

## Highlights

• Physical inactivity decreases *NR4A3* in human skeletal muscle, impacting mitochondrial function and proteostasis pathways.
• Silencing *NR4A3* reduces glucose oxidation and increases lactate production, AMPK signalling, and fatty acid oxidation.
• Downregulation of NR4A3 decreases mTORC1 signalling, ribosomal biogenesis, protein synthesis, and myotube size.
• Overexpression of NR4A3 enhances translation and cellular protein content, protecting against pharmacologically induced atrophy.

## Graphical abstract

**Figure:**
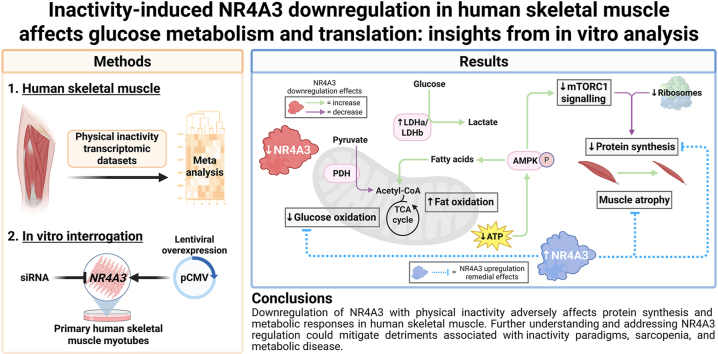

## 1 Introduction

Engagement in physical activity initiates a cascade of integrative responses related to skeletal muscle contraction [1,2], such as increased energy expenditure, haemodynamics, and oxygen consumption. Depending on the modality, intensity, and volume of exercise performed, these homeostatic processes trigger a myriad of beneficial downstream events that serve to maintain or enhance aspects of human physiology, including cardiovascular function, skeletal muscle mass, and peripheral insulin sensitivity [1,2]. Conversely, inactivity impairs skeletal muscle size and composition. These alterations have adverse effects on local tissue function and metabolism, which in turn impacts systemic health. Inactivity diminishes physical performance [3,4], whole-body metabolic flexibility [5], and is associated with reduced quality of life [6] and increased risk of non-communicable diseases like type 2 diabetes [7].

Changes in skeletal muscle mass are determined by rates of myofibrillar protein synthesis versus degradation. Physical inactivity favours signalling and transcriptional programmes that promote sustained periods of negative protein balance and muscle loss. Indeed, detectable muscle atrophy occurs within just four days of disuse [3,8]. One of the major regulators of translation is the mammalian target of rapamycin complex 1 (mTORC1), which permits myofibrillar protein synthesis and muscle hypertrophy [9], acting in tandem with ribosomal content (among other processes) to govern translation [10]. Detriments in both mTORC1 signalling [11] and ribosomal abundance [12] have been observed in skeletal muscle with inactivity. Alongside insulin- and amino acid-resistance [3,8,[13], [14], [15]], this downregulation of the translational machinery contributes towards initial losses in skeletal muscle mass through dampened myofibrillar protein synthetic responses [3,8,15]. As an effector of the phosphatidylinositol-3-kinase (PI3K)-AKT pathway, mTORC1 is also central to proteostasis via temporal coordination of anabolic and catabolic processes [16]. Nevertheless, the molecular transducers of this inactivity phenotype have not been fully elucidated.

Exercise training and physical inactivity are not mere opposing ends of a linear spectrum, but rather multifaceted processes with distinct underlying mechanisms. Understanding this dichotomy between exercise and inactivity necessitates recognising that they engage both common and distinct signalling and transcriptional pathways [17]. The NR4A families of stress-responsive orphan nuclear receptors are commonly induced by exercise [17] and all members have ties to skeletal muscle metabolism, development, or remodelling. However, only nuclear receptor subfamily 4 group A member 3 (*NR4A3*, also known as *NOR1*) shows opposite regulation in exercise and inactivity [17].

Depletion of NR4A3 reduces mitochondrial oxidative capacity and membrane potential in primary human [17] and mouse C2C12 [18] skeletal myotubes. Alternatively, transgenic overexpression of *Nr4a3* [19] or *Nr4a1* (also known as *Nur77*) [20] shifts skeletal muscle composition towards an oxidative phenotype, suggesting functional redundancy between these NR4A family members through shared NGFI-B [21] or Nur response elements [22]. Furthermore, overexpression of *Nr4a3* [19,23], *Nr4a1* [20], or *Nr4a2* (also known as *Nurr1*) [24] in skeletal muscle enhances physical performance, while deletion of *Nr4a1* impairs muscle size [25]. Silencing of *Nr4a3* in mouse C2C12 myotubes reduces mTORC1 signalling and global myosin heavy chain gene expression [18] but the molecular mechanisms underlying the metabolic, anabolic, and transcriptomic roles of NR4A3 remain largely unknown in human skeletal muscle.

Here, we investigated the impact of NR4A3 on metabolism and post-mitotic growth processes. We provide evidence that *NR4A3* is reduced during skeletal muscle disuse and that downregulation of NR4A3 impairs glucose metabolism and protein synthesis, contributing towards atrophy of skeletal muscle cells.

## 2 Methods

### 2.1 Transcriptomic meta-analysis

Transcriptomic studies of inactivity in human trials were selected using the MetaMEx database [17]. Studies were annotated with the sex and age of participants, as well as the total duration of inactivity. Statistics were performed individually for each study and the mean, variance, and *n* size were used to fit a random effects model to the data. The restricted maximum-likelihood (REML) method was applied using the R package metaphor. Obtained p-values were adjusted using the Benjamini & Hochberg method. To evaluate the effect of inactivity duration, all transcriptomic studies were merged by gene name and split into three groups: less than one week, one-two weeks, or more than two weeks. A linear model was used to test the overall effect of inactivity duration, as well as pair-wise comparisons to the pre-inactivity group, using Empirical Bayes Statistics for Differential Expression with the limma package. Gene set enrichment analysis was performed using ClusterProfiler on genes ranked based on Spearman correlation with *NR4A3*. Gene ontology biological processes were considered significant at a false discovery rate (FDR) < 0.05. The effect of reloading was estimated with the same method as above after merging GSE21496 and GSE24215.

### 2.2 Primary human cell culture

Primary cells were isolated from *vastus lateralis* skeletal muscle biopsies derived from ten healthy volunteers (age: 39 ± 16 years; BMI: 24.3 ± 1.8 kg m^−2^; mean ± SD; biological sex: ten males, two females). The regional ethical review board in Stockholm approved protocols. Cells were cultured and differentiated as described previously [26]. Successful myotube formation was monitored under the microscope and cells were used for terminal experiments ten days after the initiation of differentiation. The absence of mycoplasma contamination was routinely confirmed by PCR.

### 2.3 Silencing by RNA interference

On days six and eight of differentiation, myotubes were transfected with 10 nM of either Silencer Select siRNA Negative Control No. 2 (no. 4390847) (siScr) or Silencer Select siRNA s15542 targeting *NR4A3* (Life Technologies, Foster City, CA) (si*NR4A3*). Transfections were performed for 5 h in OptiMEM reduced serum media with Lipofectamine RNAiMAX transfection reagent (Invitrogen, Carlsbad, CA). Terminal experiments were performed ≈48 h after the second transfection.

### 2.4 Plasmid transformation, expansion, and purification

cDNA of the canonical *NR4A3* transcript (*NR4A3-203*; NM_006981.4) was synthesised into a modified pLenti CMV Puro DEST (w118-1) vector (Addgene plasmid #17452) by GENEWIZ (Azenta Life Science, Leipzig, Germany). One Shot TOP10 Chemically Competent *E. coli* cells (Invitrogen, Thermo Fisher Scientific) were used for bacterial transformation and expansion. Modified pLenti CMV Puro DEST (w118-1) empty vector (EV) and *NR4A3*-overexpressing (*NR-203*^Oex^) plasmid DNA was then purified using endotoxin-free buffers (Cat. No. 19048) and columns (Cat. No. 10083) from Qiagen, before assessing DNA concentration and purity by spectrophotometry.

### 2.5 Lentivirus production

HEK293T cells were grown in Dulbecco’s Modified Eagle Medium (DMEM; Gibco #10569) supplemented with 10% foetal bovine serum, 1% penicillin-streptomycin, and 500 μg mL^−1^ geneticin (G418). Twenty-four hours prior to transfection, HEK293T cells were seeded into T-225 flasks coated with poly-l-lysine. Once ≈90% confluent, lentiviral harvest was conducted by transfecting each T-225 with a transfection reagent mix consisting of 69 μg of EV or *NR-203*^Oex^ plasmid DNA, second generation packaging vectors (69 μg of psPAX2 DNA and 45 μg of pMD2.G DNA), 549 μg polyethylenimine (PEI; 3 μg per μg of total plasmid DNA) and 1X phosphate-buffered saline (PBS) at a final volume of 4.5 mL. The transfection mix was then added to 40.5 mL of pre-warmed growth medium (without geneticin) and added to HEK293T cells for 12–14 h. At this point, the transfection media was removed and replaced with fresh medium for 2 h before switching to new growth media supplemented with 5 mM sodium butyrate. Thirty hours later, viral media was collected and passed through a 0.45 μm low-protein binding filter (Millipore) and concentrated by low-speed centrifugation (3300 g for 30 min at 4 °C) using 100 kDa molecular weight cut-off Centricon Plus-70 cartridges (UFC710008). Three volumes of concentrated virus medium were subsequently mixed with one volume of Lenti-X concentrator (Takara Bio) and rotated at 4 °C overnight before centrifugation (1500 g for 45 min at 4 °C). Supernatant was discarded and the pellet resuspended in PBS, aliquoted, and stored at −80 °C. Virus concentration was determined using Lenti-X GoStix Plus (Takara Bio) after one freeze–thaw cycle to be consistent with experimental conditions.

### 2.6 Transduction of primary skeletal myotubes

On day 7 of differentiation, myotubes were transduced with either EV or *NR-203*^Oex^ lentivirus. Transductions were performed in high-glucose DMEM with GlutaMAX (Gibco #31966), enriched with 20% medium 199 (Gibco #31150), 2% foetal bovine serum (FBS; Sigma–Aldrich), 0.02 M HEPES buffer (Gibco #15630), 0.03 mg mL^−1^ zinc sulphate, 1.4 mg mL^−1^ vitamin B_12_ (Sigma–Aldrich), plus 100 U mL^−1^ of penicillin, 100 μg mL^−1^ of streptomycin, and 0.25 μg mL^−1^ of amphotericin B (Antibiotic-Antimycotic; Gibco #15240) (i.e. ‘post-fusion’ media). This media was then supplemented with 5 µg mL^−1^ of polybrene and purified lentivirus (concentration equivalent to 16.5 ng of p24 mL^−1^ per 9.6 cm^2^ of cells). After 18–20 h, viral medium was removed, myotubes were washed once with PBS, and then switched to normal post-fusion media [26]. Terminal experiments were performed ≈48 h later.

### 2.7 RNA extraction and analysis

RNA was isolated from cultured muscle cells using TRIzol-chloroform extraction according to manufacturer’s instruction (Invitrogen). RNA concentration and purity were determined by spectrophotometry. All equipment, software, and reagents for performing reverse transcription and RT-qPCR were from Thermo Fisher Scientific. The High-Capacity cDNA Reverse Transcription kit was used for cDNA synthesis corresponding to manufacturer’s instructions. RT-qPCR was performed on a StepOne Plus system using Taqman or SYBR Green technologies (assay ID’s and primer sequences for all analysed genes can be found in Supplementary Tables 1 and 2). Relative mRNA expression was calculated by normalising genes of interest to the geometric mean of several reference genes. The most stable reference gene combination was determined within experiments by first excluding reference genes that were statistically different between siScr vs si*NR4A3* or EV vs *NR-203*^Oex^ and/or had apparent erroneous, unstable expression behaviours after scatter plotting Ct values for each condition per donor. Next, we utilised NormFinder software, which ranks candidate reference genes or combinations thereof according to expression stability, with a cut-off threshold >0.15 [27]. Lastly, for reference gene combinations with similar NormFinder values, those with the lowest within and between donors/conditions coefficient of variation were selected, resulting in *NR4A3* silencing qPCRs being normalised to the geometric mean of *B2M*, *18S*, *TBP*, and *HPRT1*, whereas overexpression qPCRs were normalised to *B2M*, *18S*, *GUSB*, and *TBP*. Statistical analyses were performed on ΔCt values quantified in parallel using multiple t-tests or multiple Wilcoxon tests with FDR correction of 10% (q ≤ 0.1). Results were then visualised by scaling 2^−ΔCt^ values to the median 2^−ΔCt^ within a dataset of individual genes or for a relevant group of genes when primer efficiencies allowed for comparison of expression profiles (e.g. for NR4A family members). These median-scaled values were log2 transformed so that downregulation and upregulation of genes were equally represented.

### 2.8 Glucose uptake

Myotubes were washed once with PBS and incubated for 4 h in un-supplemented, serum-free low-glucose DMEM (5.5 mM glucose, Gibco #21885) (i.e. serum starvation medium). Cells were then switched to fresh serum starvation medium in the absence or presence of insulin for 1 h (120 nM; Actrapid, Novo Nordisk). Myotubes were washed once with warm glucose- and serum-free DMEM (Gibco #11966) and glucose uptake was measured in the same medium by adding 1 µL mL^−1^ of 2-[1,2–^3^H]Deoxy-d-glucose (MT911, Moravek) and 10 μM unlabelled 2-Deoxy-d-glucose for 15 min [26]. Cell monolayers were washed three times with ice-cold PBS and lysed in 1 mL 0.03% SDS. 0.5 mL of the cell lysate was counted in a liquid scintillation counter (TRI-CARB 4910 TR, PerkinElmer) and protein content of the remaining lysate was measured for normalisation of results (BCA Protein Assay Kit; #23225, Thermo Fisher Scientific, Rockford, IL).

### 2.9 Glucose incorporation into glycogen

Incorporation of glucose into glycogen was performed using similar methods detailed elsewhere [26]. Briefly, myotubes were nutrient-starved as in glucose uptake experiments and then treated with 0, 10, or 120 nM of insulin in fresh serum-starvation medium for 30 min before adding 2 μL mL^−1^ D-[^14^C(U)] glucose (NEC042B005MC; PerkinElmer) for a further 90 min. Cells were washed three times with ice-cold PBS and lysed in 0.5 mL 0.03% SDS. Thereafter, [^14^C]-labelled glycogen was extracted from 0.4 mL of the lysate and counted in a liquid scintillation counter [26]. Cellular protein concentration was determined by bicinchoninic acid (BCA) assay from the remaining 0.1 mL of cell lysate and used to normalise results.

### 2.10 Glucose oxidation

Glucose oxidation was performed using methods as described [26]. Myotubes were washed with PBS and incubated with D-[^14^C(U)] glucose in serum starvation medium in the presence or absence of 2 μM carbonyl cyanide p-trifluoro methoxyphenylhydrazone (FCCP) for 4 h. Thereafter, [^14^C]-CO_2_ was released from the medium by adding 150 μL of 2 M HCl per mL of medium and captured in 300 μL of 2 M NaOH for 1 h. The captured [^14^C]-CO_2_ was counted using a liquid scintillation counter. Cells were washed three times with ice-cold PBS and homogenised in 0.4 mL of 0.5 M NaOH. After complete lysis, 0.1 mL of 2 M HCl was added to neutralise pH and protein concentration was assessed using the BCA assay for normalisation of results.

### 2.11 Lactate assay

Lactate released into culture supernatant was measured in post-fusion medium [26] after 48 h of basal or (2 μM) FCCP-stimulated conditions using a colourimetry assay as described [26]. Assay buffer contained Tris (50 mM pH 8), NAD (7.5 mM), N-methylphenazonium methyl sulfate (250 μM), p-iodonitrotetrazolium violet (500 μM), and lactate dehydrogenase (4 U mL^−1^). Medium was passed through 3 kDa filters by centrifugation (14,000 g for 15 min at 4 °C) and 50 μL of sample was mixed with 150 μL of assay buffer. After a 20-min incubation the absorbance was read at 490 nm. Results were normalised to total cellular protein content as measured by BCA assay after lysis in 0.03% SDS.

### 2.12 Fatty acid oxidation

[^14^C(U)] palmitic acid (NEC534050UC; PerkinElmer) oxidation was performed using the same experimental procedure as glucose oxidation, except that 1 μL mL^−1^ of isotope was added to base glucose-free DMEM (Gibco #11966) supplemented with 25 μM of unlabelled BSA-conjugated palmitate.

### 2.13 Thin-layer chromatography (TLC)

Myotubes were incubated for 6 h in the same medium as described for [^14^C(U)] palmitic acid oxidation. Cells were then washed three times with ice-cold PBS and hydrophobic lipids were extracted as detailed elsewhere [28]. The resulting lipid pellet was eluted in 50 μL of a methanol:chloroform solution (1:1) and loaded on a TLC plate (Silica Gel G 250 μm 20 × 20 cm, Analtech, DE, USA). Lipid species were separated for 30 min in a loading chamber filled with 100 mL of a hexane:diethylether:acetic acid mixture (80:20:3). The loaded TLC plate was then transferred to an exposure cassette (GE Healthcare) and exposed to an X-ray film for 4 weeks at −80 °C before developing in an X-ray developer machine. Identification of the appropriate bands was determined against standards for 1,2-Dioctanyl [1–^14^C] rac-glycerol (1,2-DAG), 1,3-Dioleoyl-rac-glycerol [oleoyl-1-^14^C] (1,3-DAG), phosphatidic acid [2-palmitoyl-1-^14^C] (PA) (American Radiolabeled Chemicals Inc.), [^14^C(U)] palmitic acid (FFA), and [carboxyl-^14^C] triolein (TG) (PerkinElmer). Densitometric quantification was performed using Image Lab software (Bio-Rad) and the relative abundance of each lipid species (i.e. percentage of total detected lipids within condition) was statistically compared between groups.

### 2.14 Phenylalanine incorporation into protein

Myotubes were washed once with PBS and incubated in low-glucose base DMEM for 4 h. After starvation, media was changed to low-glucose DMEM supplemented with an additional 25 μM unlabelled phenylalanine and 4 μL of [^14^C]phenylalanine (NEC284E050UC; PerkinElmer) per mL for 6 h, with or without 20% FBS plus 10 mM leucine, and in the presence or absence of 100 nM rapamycin or 10 μM lactacystin. Cells were then washed three times with ice-cold PBS and lysed in 0.5 mL 0.03% SDS. Protein concentration was measured from 0.1 mL of cell lysate by BCA protein assay. Protein from the remaining cell lysate was precipitated in 50% Trichloroacetic acid with 1% BSA overnight at −20 °C, followed by centrifugation (12,000 g for 15 min at 4 °C). Supernatant was discarded and the protein pellet washed twice in acetone with centrifugation after each wash (12,000 g for 15 min at 4 °C). Acetone was discarded and the protein pellet dissolved in 0.5 M NaOH for 1 h in a heating block at 65 °C with shaking. The dissolved pellet homogenate was transferred to scintillation vials and the amount of [^14^C] phenylalanine incorporation determined by scintillation counting.

### 2.15 Surface sensing of translation (SUnSET) assay

Relative rates of *in vitro* protein synthesis were determined using the SUnSET method as described [29]. Briefly, myotubes were washed once in PBS and incubated in low-glucose base DMEM for 4 h. Media was then changed to fresh low-glucose vehicle DMEM or low-glucose DMEM supplemented with 120 nM insulin and 10 mM leucine, with or without 100 nM rapamycin or 178 μM cycloheximide (*NR-203*^Oex^ experiment only). After 1 h of treatment, puromycin (1 μM) was added to the medium and cells were incubated for a further 30 min before collection. Medium was aspirated and cells were washed once in ice-cold PBS. Samples were then harvested in protein lysis buffer (137 mM NaCl, 2.7 mM KCl, 1 mM MgCl_2_, 1% Triton X-100, 10% glycerol, 20 mM Tris pH 7.8, 10 mM NaF, 1 mM EDTA, 1 mM PMSF, 0.5 mM Na_3_VO_4_ and 1x PIC), rotated for 30 min at 4 °C and subjected to centrifugation (12,000 g for 15 min at 4 °C). Supernatants were stored at −80 °C until protein determination by BCA assay and subsequent sample preparation for immunoblotting.

### 2.16 Insulin stimulation for assessment of signalling

For interrogation of the canonical insulin signalling cascade, myotubes were incubated in serum starvation medium for 4 h. Cells were then stimulated with 0, 10, or 120 nM of insulin in fresh serum starvation medium for 20 min and lysed as mentioned for SUnSET assay samples.

### 2.17 Immunoblot analysis

Samples were prepared for SDS-PAGE with Laemmli buffer (60 mM Tris pH 6.8, 2% w/v SDS, 10% v/v glycerol, 0.01% w/v bromophenol blue, 1.25% v/v β-mercaptoethanol). Equal amounts of protein were loaded and separated on Criterion XT 4–12% Bis-Tris Gels (Bio-Rad, Hercules, CA) and transferred to polyvinylidene fluoride membranes (Merck, Germany). Membranes were stained with Ponceau S to confirm transfer quality and to ensure equal loading of lanes for each gel run prior to quantifying targets of interest. Membranes were then blocked with 5% non-fat milk in Tris-buffered saline supplemented with Tween-20 (TBST; 20 mM tris–HCl pH 7.6, 137 mM NaCl, 0.02% Tween-20) for 1 h at room temperature, before an overnight incubation with primary antibodies at 4 °C with gentle rocking. Primary antibodies were diluted in TBS plus 0.1% w/v bovine serum albumin and 0.1% w/v NaN_3_, and specific antibodies, as well as dilutions used, can be found in Supplementary Table 3. Membranes were next washed with TBST and incubated with species-appropriate horseradish peroxidase-conjugated secondary antibody (1:25,000 in TBST with 5% non-fat milk). Proteins were visualised by enhanced chemiluminescence (Amersham ECL Western Blotting Detection Reagent, UK) and quantified by densitometry (QuantityOne or Image Lab software, Bio-Rad). Subsequent statistical analyses were conducted on densitometries scaled to the median value for each phosphorylation event, total protein abundance, or phosphorylated-to-total protein ratio assessed. Median-scaled densitometries were then plotted on a log2 scale for equivalent representation of decreases and increases within measured signalling events.

### 2.18 Total cellular protein and RNA

Unless stated otherwise, total protein concentrations reported in figures are from BCA protein assay measurements of basal conditions in metabolic assays. Compared to sample lysis for immunoblot analysis, homogenisation of cell monolayers for normalisation of metabolic assays was performed in larger volumes of buffer. Thus, these measurements were less affected by small losses in lysate volume and/or pipetting errors and more accurately reflected total protein concentration per well. In cases where total protein, total RNA, or select target protein levels (i.e. immunoblotting) were determined in multiple independent experiments using primary cells from the same donor, values from each experiment were log2 transformed, Z-scored, and the average Z-score across assays was used for statistical analysis and visualisation of results.

### 2.19 Immunocytochemistry

Myotubes were immuno-stained for desmin (DES, 1:500, Ab15200, Abcam) or fast myosin heavy chain isoforms IIA and IIX (MyHC-IIA/IIX) (MYH1/2, 1:250; sc-53088, Santa Cruz Biotechnology) in 6-well culturing plates using methods as described [26], with the addition of nuclear counterstaining. After 90 min incubation with Alexa Fluor 488 goat anti-rabbit (A-11008) or Alexa Fluor 594 goat anti-mouse (A-11005) secondary antibodies (1:500, Thermo Fisher Scientific) at room temperature with gentle rocking, cells were washed twice with 0.025% Tween-20 in PBS and counterstained with 300 nM 4′,6-diamidino-2-phenylindole (DAPI) in PBS for 5 min at room temperature. Myotubes were then washed twice in PBS and kept in PBS protected from light at 4 °C until imaging. DES, MyHC-IIA/IIX, and nuclei images were taken using 20× magnification on a Zeiss Axio Vert.A1 inverted fluorescent microscope, equipped with ZEN software (Carl Zeiss Microscopy GmbH, Germany). Images were obtained from 3 to 5 random fields of view per well across three technical replicate wells, producing 9–13 images per condition for each donor. Files were subsequently uploaded to ImageJ (Fiji) and their local contrast enhanced using the Contrast Limited Adaptive Histogram Equalization (CLAHE) plugin. For DES and MyHC-IIA/IIX quantification, image masks were created and smoothed after colour threshold adjustment. Myotube area (μm^2^) per field was then measured using the Analyse Pixels command with a pixel-threshold ≥50 μm^2^. Nuclei were counted manually using the ImageJ cell-counter plugin. Fused myonuclei were considered nuclei counted within the MyHC-IIA/IIX area automatically detected by ImageJ software in the MyHC-IIA/IIX analysis and fusion index calculated as the percentage of fused versus total nuclei per field of view. EV and *NR-203*^Oex^ transduced muscle cells were cultured for ≈48 h in the presence or absence of 10 μM of dexamethasone prior to fixation for immunostaining.

### 2.20 Statistics

Analyses were performed using either R 4.1.0 (www.r-project.org) or GraphPad Prism 10.0.3 software (GraphPad Software Inc.). For transparency of statistics, exact p-values are specified for comparisons where probability met *p* < 0.1. Differences were considered significant at p ≤ 0.05, except when performing multiple comparisons of p ≤ 0.05 main/interaction effects in 3-way ANOVAs and for multiple t-test analysis of qPCR results, where FDR correction was used with an *a priori* threshold set at 10% (q ≤ 0.1). Data are presented as box-and-whisker plots with Tukey distribution and pairing by donor unless specified otherwise in figure legends. Normality was assessed using Shapiro–Wilk test before applying appropriate parametric or non-parametric tests. In 2-way and 3-way ANOVA analyses, Tukey ladder transformation was used when residuals violated assumptions of normality. Statistical tests and sample sizes are described in figure legends. Bioinformatic, qPCR, and immunoblot analyses are described in their respective method sections.

## 3 Results

### 3.1 Downregulation of *NR4A3* during inactivity is associated with remodelling of myogenic and metabolic pathways

*NR4A3* mRNA is induced by exercise and repressed during inactivity in human skeletal muscle [17]. We previously reported that gene expression profiles of primary human skeletal myotubes subjected to *NR4A3* silencing best reflects the human muscle transcriptome after periods of bedrest and limb immobilisation [17]. To build upon this observation and gain further insight into the regulation of *NR4A3*, we analysed eight published transcriptomic studies of the human skeletal muscle response to inactivity.

**Figure 1:**
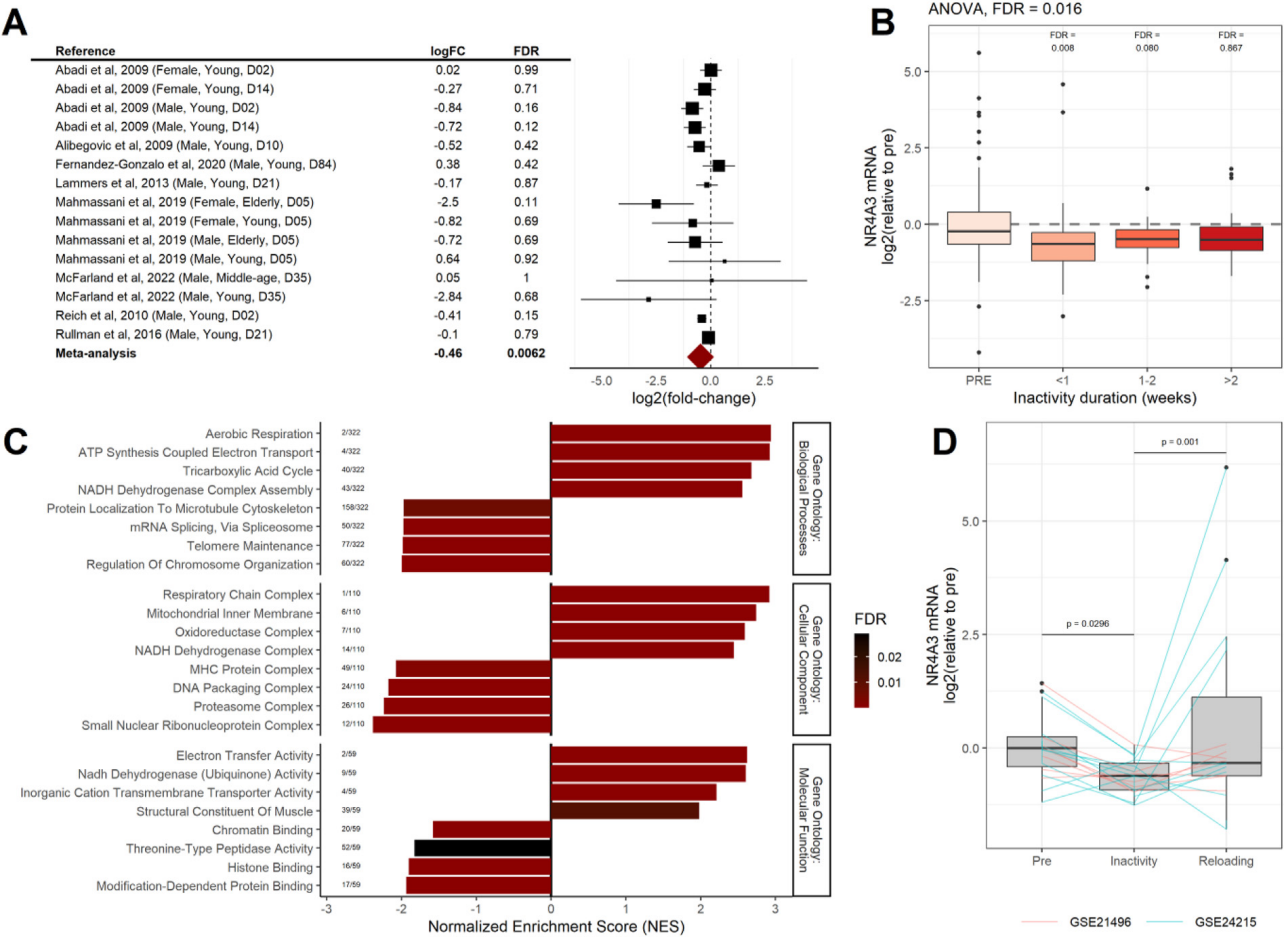
**Downregulation of *NR4A3* during inactivity is associated with remodelling of myogenic and metabolic pathways. A.** Meta-analysis of transcriptomic inactivity studies in human skeletal muscle. **B.** Transcriptomic studies were pooled by the duration of inactivity protocols: less than one week, one-two weeks, or more than two weeks. Datasets were normalised as described in methods. **C.** Gene set enrichment analysis with the Gene Ontology dataset was performed on genes ranked on Spearman correlation with *NR4A3* across all transcriptomic studies of inactivity. Bar colour represents FDR and numbers to the left of each bar indicate the rank of each ontology within the total number of enriched pathways. **D.** The two studies that explored effects of reloading after inactivity were merged and analysed as described in methods. (For interpretation of the references to colour in this figure legend, the reader is referred to the Web version of this article.)

This meta-analysis revealed that physical inactivity reduced *NR4A3* mRNA by 27% (95% CI: 42, 9), but this decrease was not evident in all studies (Figure 1A). Grouping studies based on the duration of inactivity demonstrated that skeletal muscle *NR4A3* expression is transiently repressed during the first days of disuse before returning to baseline levels after 2 weeks (Figure 1B). Correlation of *NR4A3* with all other transcripts present in the eight studies allowed for the identification of pathways co-regulated with *NR4A3* during inactivity (Figure 1C). Gene set enrichment analysis showed that *NR4A3* was positively associated with mitochondrial function and negatively associated with pathways related to cytoskeleton organisation, chromatin regulation, protein synthesis, and degradation (Figure 1C). Furthermore, combined analysis of two datasets exploring the restoration of ambulatory behaviour after inactivity showed that reloading re-established *NR4A3* mRNA to pre-inactivity levels (Figure 1D), consistent with its role as a contraction-responsive transcription factor in skeletal muscle [17,23,30].

### 3.2 Silencing *NR4A3* attenuates glucose oxidation by diverting glucose towards lactate and increases fatty acid oxidation

**Figure 2:**
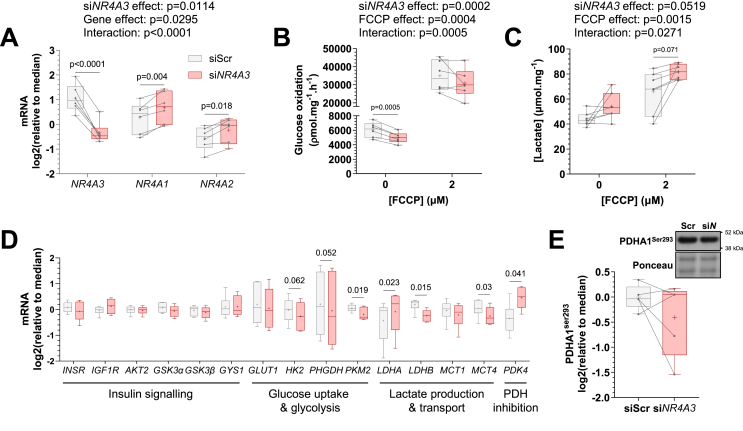
***NR4A3* silencing in primary human myotubes attenuates glucose oxidation by diverting glucose towards lactate production.** Primary skeletal muscle cells were exposed to a control scramble sequence (siScr) or a silencing RNA targeting *NR4A3* (si*NR4A3*). **A.** mRNA expression of NR4A family members *NR4A3*, *NR4A1* and *NR4A2* measured by RT-qPCR. *n* = 6, 2-way ANOVA (silencing x gene) with Šidák correction. **B.** Rates of radiolabelled glucose oxidation under basal and (2 μM) FCCP-stimulated conditions over 4 h. *n* = 6, 2-way ANOVA (silencing x FCCP) with Šidák correction. **C.** Lactate concentration in cell supernatant measured after 48 h of basal or (2 μM) FCCP-stimulated conditions. *n* = 7, 2-way ANOVA (silencing x FCCP) with Šidák correction. Overall statistical model effects are stated in figures. **D.** mRNA expression of genes involved in glucose metabolism measured by RT-qPCR. Results are box-and-whisker plots with Tukey distribution and crosses indicating mean values. *n* = 6, multiple paired t-tests with FDR correction. **E.** Representative immunoblot and quantification of pyruvate dehydrogenase E1 Subunit Alpha 1 (PDHA1) phosphorylation at Ser^293^. *n* = 5, 2-way ANOVA (silencing x insulin + leucine). Only basal data is shown.

To mimic the decreased levels observed in human skeletal muscle after inactivity, *NR4A3* was experimentally downregulated using RNA interference in differentiated primary human skeletal muscle cells. Silencing of *NR4A3* was associated with modest compensatory increases in other NR4A family members *NR4A1* (*NUR77*) and *NR4A2* (*NURR1*) (Figure 2A). Importantly, depletion of *NR4A3* lowered basal and FCCP-stimulated (uncoupled) glucose oxidation (Figure 2B) independent from changes in glucose transport. Rather, several phosphorylation events in the canonical insulin signalling cascade were potentiated by silencing of *NR4A3* (Supplementary Fig. 1), including site Thr308 on AKT and downstream inactivating phosphorylation of glycogen synthase kinase 3 (GSK3) α and β. Furthermore, basal and insulin-mediated glucose uptake and incorporation into glycogen responses were maintained (Supplementary Figures 1H, 1I).

Upon *NR4A3* silencing, lactate production and release into culture medium was augmented (Figure 2C), indicating a shift in glucose fate that was also reported in mouse C2C12 myotubes [33]. Accordingly, mRNA expression of the alpha isoform of lactate dehydrogenase (*LDHA*) increased after *NR4A3* silencing, while expression of the beta isoform decreased (*LDHB*) (Figure 2D). While total LDH protein abundance remained unaltered (Supplementary Fig. 1K), the modification of LDH isoform composition indicates preferential production of lactate from glycolysis and/or less conversion of lactate to pyruvate [34,35]. This notion was further supported by the reduction of pyruvate kinase muscle 2 (*PKM2*) mRNA (Figure 2D), as inhibiting noncanonical PKM2 activity increased lactate release from C2C12 myoblasts [36]. For glucose to be oxidised in the mitochondria, pyruvate must be metabolised to acetyl coenzyme A (acetyl-CoA) via the pyruvate dehydrogenase (PDH) complex. *NR4A3* silencing upregulated pyruvate dehydrogenase kinase 4 (*PDK4*) gene expression (Figure 2D) compatible with attenuated glucose oxidation [37] (Figure 2B). However, the inhibitory PDK4-target phosphorylation site on PDH E1 component subunit alpha (PDHA1^Ser293^) was unaltered (Figure 2E), indicating that overall PDH activity was not perturbed.

We previously reported that mitochondrial respiration (in the presence of glucose, glutamine, and pyruvate) and protein subunits of the electron transport chain were diminished by *NR4A3* silencing in primary human skeletal myotubes [17]. This aligns with findings in C2C12 myotubes, where *Nr4a3* depletion decreased mitochondrial membrane potential in the absence of changes in mitochondrial DNA [18]. Instead, downregulation of *Nr4a3* negatively affected the expression of respiratory complex genes, as well as transcripts for mitochondrial ribosomal proteins [18]. Additionally, *Nr4a3* silencing reduced the mRNA abundance of mitofusin-2 (*Mfn2*) but increased dynamin 1 like protein expression (DNM1L; also known as DRP1) [18]. These data suggest that loss of NR4A3 attenuates mitochondrial fusion dynamics (instead favouring fission), mitochondrial reticulum connectivity, and assembly of electron transport chain complexes in a manner separate from alterations in mitochondrial abundance. Our finding that myotube response to the mitochondrial uncoupler FCCP was maintained after *NR4A3* RNA interference further supports not only retained mitochondrial content, but also the presence of functional mitochondria (Figure 2B). Thus, the attenuation of glucose oxidation in NR4A3-depleted myotubes appears to be a consequence of greater diversion of glucose towards lactate, which would reduce glucose-derived acetyl-CoA entry into the tricarboxylic acid (TCA) cycle.

**Figure 3:**
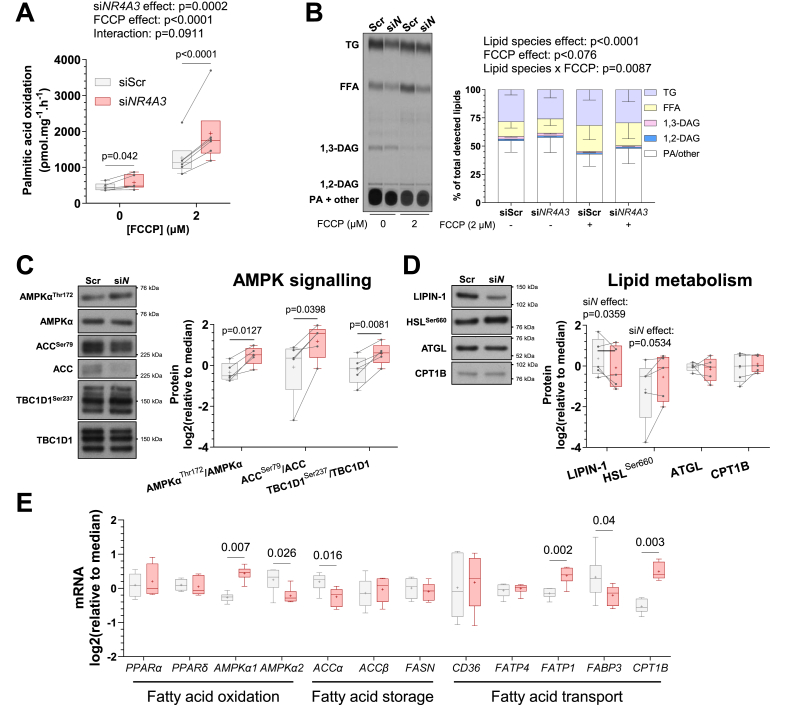
***NR4A3* silencing upregulates fatty acid oxidation and AMPK signalling.** Primary skeletal muscle cells were exposed to a control scramble sequence (siScr) or a silencing RNA targeting *NR4A3* (si*NR4A3*). **A.** Rates of radiolabelled ^14^C palmitic acid oxidation under basal and (2 μM) FCCP-stimulated conditions over 4 h. *n* = 6, 2-way ANOVA (silencing x FCCP) with Šidák correction. **B.** Detection of select lipid species by thin-layer chromatography (TLC) of lipid extracts from myotubes after 6 h of basal or (2 μM) FCCP-stimulated conditions. TG = triacylglycerols, FFA = free fatty acids, DAG = diacylglycerols, PA + other = phosphatidic acid plus all other non-migrating lipid species. Results are the mean ± SEM of each lipid species as a percentage of total detected lipid species within condition. *n* = 5, 3-way ANOVA (silencing x lipid species x FCCP). Overall statistical model effects are stated in figures. **C.** Immunoblot of AMPK-signalling proteins from an indicative donor and quantification of phosphorylated-to-total protein ratios. *n* = 5, 2-way ANOVA (silencing x insulin + leucine) with Šidák correction. Only basal data are shown. Analysis of phospho- and total protein levels can be found in Supplementary Fig. 2. **D.** Representative immunoblot and quantification of proteins involved in lipid storage, mobilisation, and transport. *n* = 5, 2-way ANOVA (silencing x insulin + leucine) with Šidák correction. Only basal data are shown and overall effects of silencing in the model are presented (si*N* effect, *p* < 0.1). **E.** mRNA expression of genes involved in lipid metabolism measured by RT-qPCR. Results are box-and-whisker plots with Tukey distribution and crosses indicating mean values. *n* = 6, multiple paired t-tests with FDR correction.

Skeletal muscle cells are highly glycolytic in culture, preferentially oxidising glucose at rates 100-fold faster than long-chain fatty acids [26]. Therefore, we explored the consequence of NR4A3-dependent reductions in glucose oxidation on lipid metabolism in primary human skeletal myotubes. *NR4A3* depletion upregulated basal and FCCP-stimulated rates of fatty acid oxidation (Figure 3A) without altering the intracellular composition of select lipid species. Whereas the ratio of triacylglycerols, diacylglycerols, free fatty acids, and other lipids were modified by FCCP, *NR4A3* RNA interference was without effect (Figure 3B). Compatible with greater fatty acid oxidation, *NR4A3* silencing increased phosphorylation of the AMP-activated protein kinase α subunit (AMPKα^Thr172^) and its substrates acetyl-CoA carboxylase (ACC^Ser79^) and TBC domain family member 1 (TBC1D1^Ser237^) (Figure 3C; phosphoprotein and total protein levels are depicted separately in Supplementary Fig. 2A–C). This signalling coalesced with activating phosphorylation of hormone-sensitive lipase (HSL^Ser660^), reduced LIPIN1 protein abundance (Figure 3D), and downregulation of total ACC protein (Figure 3C; Supplementary Fig. 2B), as well as the mRNA of its cytosolic isoform (*ACCα*) (Figure 3E). Further profiling of transcripts involved in fatty acid metabolic pathways revealed that NR4A3 depletion altered the composition of *AMPKα* isoforms and fatty acid transport genes (Figure 3E). Compared to control, *NR4A3* silencing increased the expression of fatty acid transport protein 1 (*FATP1*) and reduced the expression of fatty acid binding protein 3 (*FABP3*) (Figure 3E). Together, the protein and transcriptional changes induced after RNA interference of *NR4A3* suggest a physiological mechanism compensating for energy stress due to impaired glucose oxidation.

### 3.3 *NR4A3* silencing in primary human myotubes downregulates translation, mTORC1 signalling, and pre-rRNA abundance

**Figure 4:**
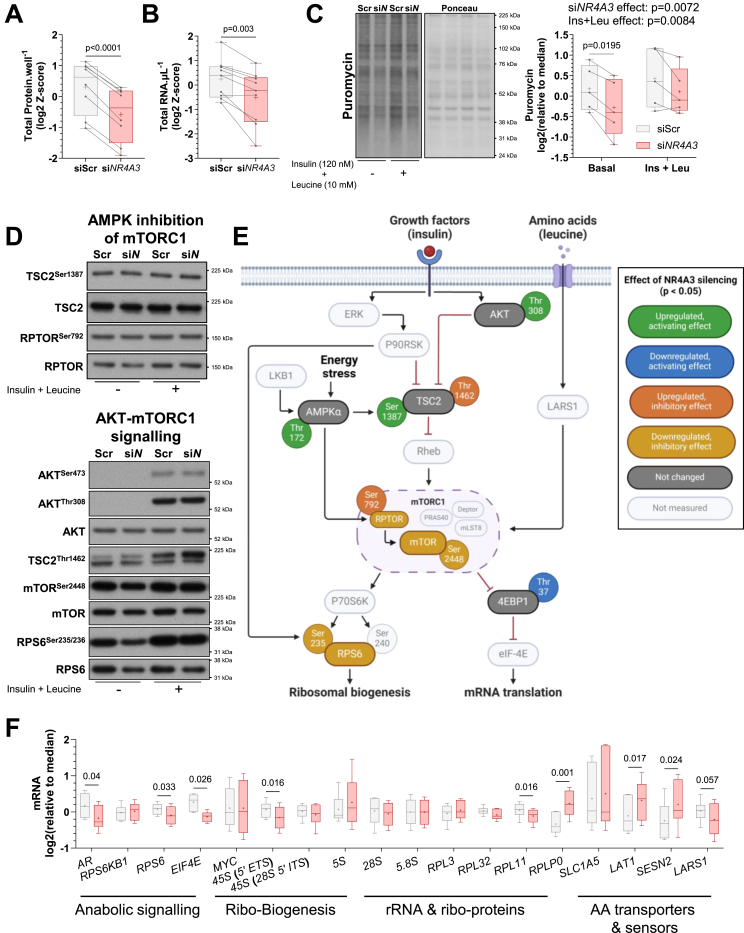
***NR4A3* silencing impairs mTORC1 signalling, ribosomal biogenesis, and translation.** Primary skeletal muscle cells were exposed to a control scramble sequence (siScr) or a silencing RNA targeting *NR4A3* (si*NR4A3*). **A.** Total protein per well and **B.** RNA per μL. Results are the average Z-score of log2-transformed values across experiments. *n* = 8 for protein and *n* = 9 for RNA, paired t-test. **C.** Representative immunoblot and quantification of cellular protein synthesis assessed by protein puromycilation (i.e. SUnSET method). *n* = 5, 2-way ANOVA (silencing x insulin + leucine) with Šidák correction. Overall statistical model effects are stated in figure. **D.** Immunoblot of proteins involved in AMPK-mediated mTORC1 inhibition and AKT-mTORC1 pathways from an indicative donor. **E.** Schematic representation of immunoblot analysis. *n* = 5, 2-way ANOVA (silencing x insulin + leucine) with Šidák correction. Quantification of each depicted signalling event can be found in Supplementary Fig. 3. **F.** mRNA expression of genes involved in amino acid (AA) metabolism and protein synthesis measured by RT-qPCR. Results are box-and-whisker plots with Tukey distribution and crosses indicating mean values. *n* = 6, multiple paired t-tests or Wilcoxon signed-rank tests with FDR correction. 45S = 45S pre-rRNA, 5′ ETS and ITS = 5-prime external and internal transcribed spacers, ‘Ribo-’ = ribosomal.

Metabolic assays are commonly normalised to protein content to account for differences in seeding density. We observed that silencing of *NR4A3* robustly decreased the total protein and RNA abundance of cultured human myotubes (Figure 4A, B). These findings, combined with the association of reduced *NR4A3* levels during bed rest and limb immobilisation inactivity (Figure 1), led us to explore the putative effects of *NR4A3* silencing on protein synthesis. Relative rates of translation can be measured *in vitro* by the surface sensing of translation (SUnSET) method, which detects puromycin incorporation into nascent peptide chains by immunoblot analysis [29]. Using this technique, we observed attenuated puromycilation of proteins upon *NR4A3* silencing both at baseline and after insulin plus leucine stimulation (Figure 4C), suggesting impaired protein synthesis with NR4A3 downregulation.

The efficiency and total capacity for translation in skeletal muscle is determined by mTORC1 signalling and ribosomal content, respectively [10,12]. *In vitro*, AMPK impedes mTORC1 activity through phosphorylation of tuberous sclerosis complex 2 (TSC2) [16,38] and the regulatory-associated protein of mTOR (RPTOR) [16,39]. Consistent with energy-stress dependent upregulation of AMPK (Figure 3C), the ratio of phosphorylated-to-total protein of AMPK target sites on TSC2 (Ser1387) and RPTOR (Ser792) were increased by NR4A3 depletion (Figure 4D–E; Supplementary Fig. 3A) indicative of AMPK interference of mTORC1. Furthermore, *NR4A3* silencing reduced the protein abundance of RPTOR, mTOR, and ribosomal protein S6 (RPS6) (Figure 4D–E; Supplementary Fig. 3B–D). These collective AMPK-related and unrelated changes coalesced to inhibit mTOR^Ser2448^ phosphorylation and activation of downstream mTORC1-substrates, including RPS6^Ser235/236^ and eukaryotic translation initiation factor 4E-binding protein 1 (4EBP1) Thr37/46 phosphorylation, with negative consequences for skeletal muscle protein synthesis following NR4A3 depletion (Figure 4D, E; Supplementary Fig. 3C–E).

In agreement with diminished translation, gene expression analyses confirmed that *NR4A3* RNA interference decreased mRNA abundance of *RPS6*, as well as eukaryotic translation initiation factor 4E (*EIF4E*) and 45S pre-ribosomal RNA (5-prime external transcribed spacer; *5′ ETS*) (Figure 4F). The 45S pre-rRNA polycistronic precursor is transcribed by RNA polymerase I (Pol I) and gives rise to the 18S, 5.8S, and 28S rRNAs. These rRNAs are subsequently assembled with Pol II-dependent ribosomal proteins and Pol III-transcribed 5S rRNA into the functional 40S (small) and 60S (large) ribosomal subunits [40]. As such, Pol I activity is the rate-limiting step in ribosomal biogenesis. Due to the short half-life of the ETS region [41], measurement of the 45S pre-RNA 5′ ETS is a reliable indicator of Pol I-mediated ribosomal transcription [40]. Thus, our results imply that lower levels of total RNA from *NR4A3*-silenced myotubes (Figure 4B) are a consequence of attenuated ribosomal biogenesis, resulting in reduced ribosomal mass, and that NR4A3 downregulation impedes both translational efficiency (i.e. mTORC1) and capacity (i.e. ribosomal abundance).

Despite an overall reduction in protein synthesis (Figure 4C), NR4A3-depleted myotubes still responded to insulin plus leucine stimulation, as suggested by the interaction between *NR4A3* silencing and treatment for mTOR^Ser2488^, RPS6^Ser235/236^, and the inhibitory AKT-target phosphorylation site on TSC2 (Thr1462) (Figure 4D; Supplementary Figures 3C, D, F). AKT^Thr308^ levels were greater upon insulin treatment (Supplementary Figures 1A, E) and tended to increase with insulin plus leucine stimulation (Figure 4D–E; Supplementary Fig. 3G). Furthermore, mRNA of the large neutral amino acid transporter small subunit 1 (*LAT1*; also known as *SLC7A5*) was upregulated by *NR4A3* silencing (Figure 4F). LAT1 is the dominant antiporter for leucine uptake into cells [42]. The rise in intracellular leucine is then detected through biochemical sensing mechanisms, including disassociation of sestrin-2 (SESN2) with GATOR2 [16,43] and activation of leucyl-tRNA synthetase 1 (LARS1) [44], with subsequent initiation of mTORC1. As such, increased LAT1 could feasibly support higher intramuscular leucine concentrations under *NR4A3*-silenced conditions and somewhat offset the detrimental effects of increased *SESN2* or reduced *LARS1* mRNA (Figure 4F). Hence, canonical insulin signalling, combined with enhanced leucine transport, provides a mechanism by which anabolic sensitivity of NR4A3-depleted myotubes is at least partially retained via the AKT-mTORC1 pathway.

We next investigated the impact of NR4A3 downregulation on markers of inflammation and protein degradation. Although the contribution of protein breakdown towards human disuse muscle atrophy is unclear [3,8,15], the calpain, caspase, ubiquitin-proteasomal (UPS) and autophagy-lysosomal systems are markedly induced during murine models of inactivity [45]. NR4A3 depletion upregulated activating transcription factor 3 (*ATF3*) and DNA damage-inducible transcript 3 (*DDIT3*; also known as *CHOP*) mRNA, whilst reducing IkBα protein abundance (Supplementary Fig. 4A–C), signifying possible inflammation-mediated sarcoplasmic reticulum stress. However, other surrogates of inflammatory nuclear factor kappa B (NF-kB) signalling and the unfolded protein response, such as interleukin 6 (*IL-6*) and *ATF4*, were unaltered (Supplementary Fig. 4A).

Major proteolytic pathways use ubiquitination as a signal for select protein degradation. Indeed, overexpression of the muscle-specific E3 ubiquitin ligase muscle ring-finger 1 (*MuRF1* or *Trim63*) is sufficient to induce ubiquitination and muscle atrophy in mice [46]. The *MuRF1* promoter is a direct target of forkhead box O3 (FOXO3a) [47] and inhibitory phosphorylation of FOXO3a and FOXO1 were lower after *NR4A3* interference (Supplementary Fig. 4D–E), indicative of greater FOXO transcriptional activity. Accordingly, mRNA expression of *MuRF1* and *FOXO3* were also elevated (Supplementary Fig. 4A). However, these findings were contrasted by downregulation of both MuRF1 and FOXO3a protein levels, while FOXO1 and the alternative muscle-enriched E3 ligase MAFbx (also known as atrogin-1) were unperturbed (Supplementary Fig. 4D–G). Furthermore, contrary to the stark impairment of protein synthesis, *NR4A3* silencing did not alter global protein ubiquitination in primary skeletal myotubes (Supplementary Fig. 4H). Rather, the proteases calpain-1 (including the activated, autolysed form) and caspase 3 (inactive zymogen form; autolysed form undetected) were decreased upon NR4A3 depletion, as were alpha subunit proteins of the 20S core particle proteasome (pan-20Sα) (Supplementary Fig. 4I–K), whereas proteasome 20S subunit alpha 1 (*PSMA1*) and proteasome 20S subunit beta 2 (*PSMB2*) transcripts were induced and attenuated, respectively (Supplementary Fig. 4A). In addition, despite adeno-associated virus (AAV) [23] and skeletal muscle-specific transgenic [48] *Nr4a3* gain-of-function mice displaying greater autophagic flux, *NR4A3* interference had no impact on autophagy markers unc-51 like autophagy activating kinase 1 (ULK1), ubiquitin-binding protein p62 (also known as sequestrosome-1), or microtubule-associated proteins 1A/1B-light chain 3 (LC3-I and LC3-II) (Supplementary Figures 4L-N).

Altogether, silencing *NR4A3* in skeletal myotubes triggered AMPK-dependent and independent inhibition of mTORC1, reducing ribosomal biogenesis and protein translation. Pathways related to proteolysis were negligibly affected; suggesting NR4A3 downregulation primarily reduces skeletal muscle protein content via multi-level attenuation of protein synthesis.

### 3.4 Overexpression of the canonical *NR4A3* isoform increases protein synthesis in primary human skeletal myotubes

**Figure 5:**
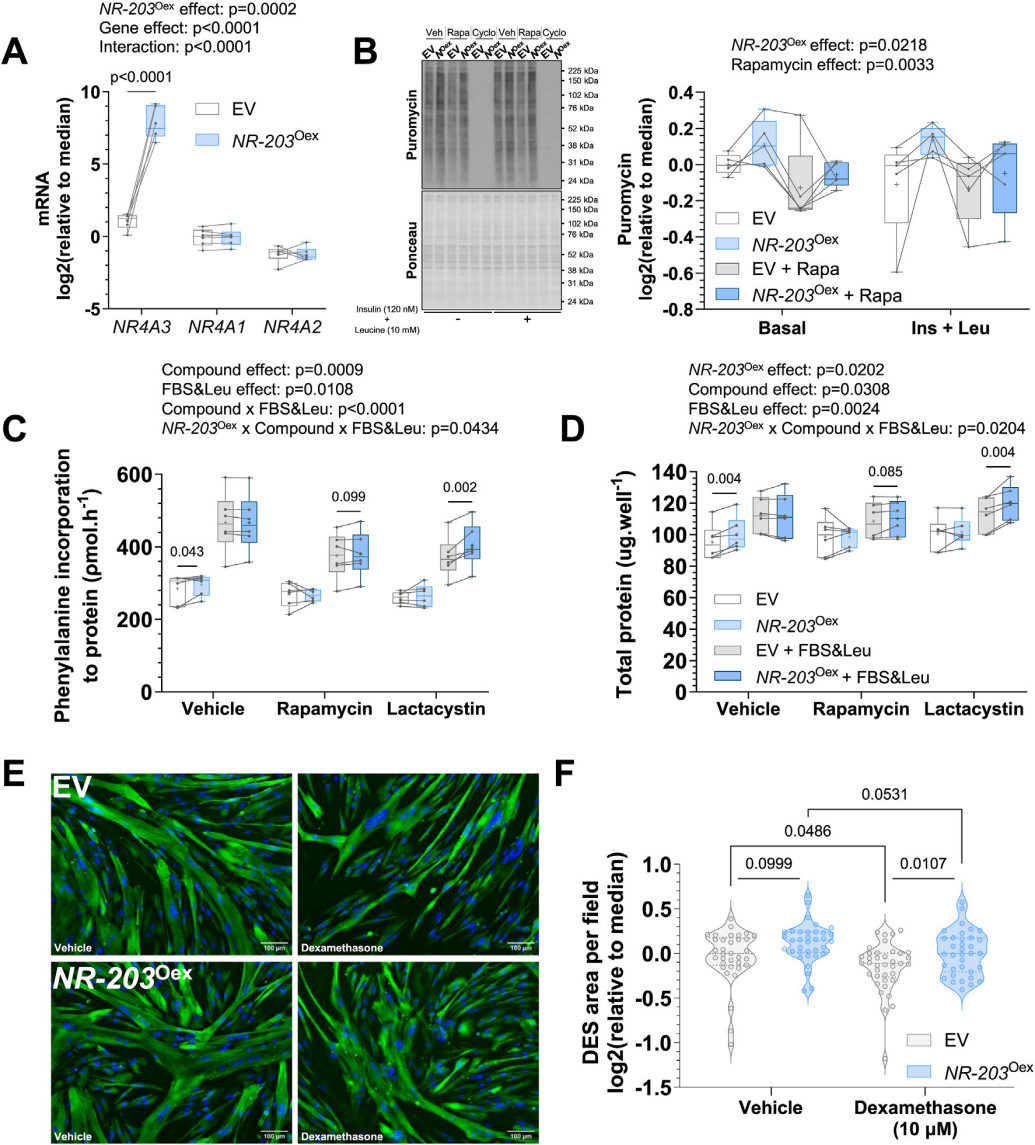
**Overexpression of the canonical *NR4A3-203* isoform increases protein synthesis and protects against dexamethasone-induced myotube atrophy.** Primary skeletal muscle cells were transduced with an empty vector control plasmid (EV) or a plasmid containing variant *NR4A3-203* (*NR-203*^Oex^). **A.** mRNA expression of NR4A family members *NR4A3*, *NR4A1* and *NR4A2* measured by RT-qPCR. *n* = 6, 2-way ANOVA (overexpression x gene) with Šidák correction. **B.** Indicative immunoblot and quantification of cellular protein synthesis assessed by protein puromycilation (i.e. SUnSET method). *n* = 5, 3-way ANOVA (overexpression x rapamycin x insulin + leucine) with FDR correction. Veh = vehicle, Rapa = rapamycin, and Cyclo = cycloheximide treatments, respectively. **C.** Rates of radiolabelled phenylalanine incorporation to protein under basal and (20%) FBS plus (10 mM) leucine-stimulated conditions over 6 h, with or without mTORC1- (rapamycin) or proteasomal- (lactacystin) inhibition. *n* = 6, 3-way ANOVA (overexpression x FBS + Leucine x compound) with FDR correction. **D.** Total protein per well from same experiment as C. Overall statistical model effects are stated in figures. **E.** Immunocytochemistry of desmin (DES, green) and nuclei (DAPI, blue) from a representative donor. Scale bar = 100 μm. **F.** Quantification of immunocytochemistry for DES area per field of view, with or without 48 h of dexamethasone treatment. Results are violin plots with median and interquartile range. Circles represent measurements from different fields of view across three technical replicates per donor. *n* = 3, nested paired t-tests. (For interpretation of the references to colour in this figure legend, the reader is referred to the Web version of this article.)

In humans, the *NR4A3* gene undergoes alternative splicing [31,49], producing four transcripts [50] that encode for three isoforms of the NR4A3 protein: two longer variants, which differ by 11 amino acids in their N-terminus, and a truncated variant containing a distinct C-terminus that lacks the ligand binding domain [49] (UniProtKB Q92570 [32]). The RNA interference method described in previous figures targeted an exon common to all isoforms, precluding the analysis of differential effects of select *NR4A3* variants. Thus, we explored lentiviral overexpression of the canonical *NR4A3* isoform (*NR4A3-203*; NM_006981.4) in primary human skeletal myotubes. Overexpression of the *NR4A3-203* (*NR-203*) transcript increased total *NR4A3* mRNA levels by > 140-fold without altering the abundance of other NR4A family members (Figure 5A) or perturbing aspects of glucose and fatty acid metabolism (Supplementary Fig. 5A–D). However, congruous with the effects of *NR4A3* silencing, *NR4A3-203* overexpression enhanced protein synthesis at baseline and after insulin plus leucine treatment (Figure 5B). This result was coherent with hypertrophy of mouse muscle overexpressing *Nr4a3* [48] and further supported by assessment of radiolabelled phenylalanine incorporation into protein. Here, canonical *NR4A3* overexpression increased rates of translation under both unstimulated conditions and after foetal bovine serum (FBS) plus leucine treatment when combined with proteasomal blockade (lactacystin) or mTORC1-inhibition (rapamycin) (Figure 5C). That total cellular protein content of *NR4A3-203* overexpressing myotubes was greater in these same conditions implies the canonical NR4A3 isoform may upregulate overall protein turnover in a manner partly independent from mTORC1 (Figure 5D). Indeed, no differences in total RNA concentrations or mTORC1 signalling were observed after *NR4A3-203* overexpression (Supplementary Figures 5E, 5F), suggesting that *NR4A3* silencing and overexpression phenotypes manifest through different mechanisms. This hypothesis was further substantiated by the failure of NR4A3-203 overexpression to modulate transcripts affected by silencing (Supplementary Fig. 5G).

**Figure 6:**
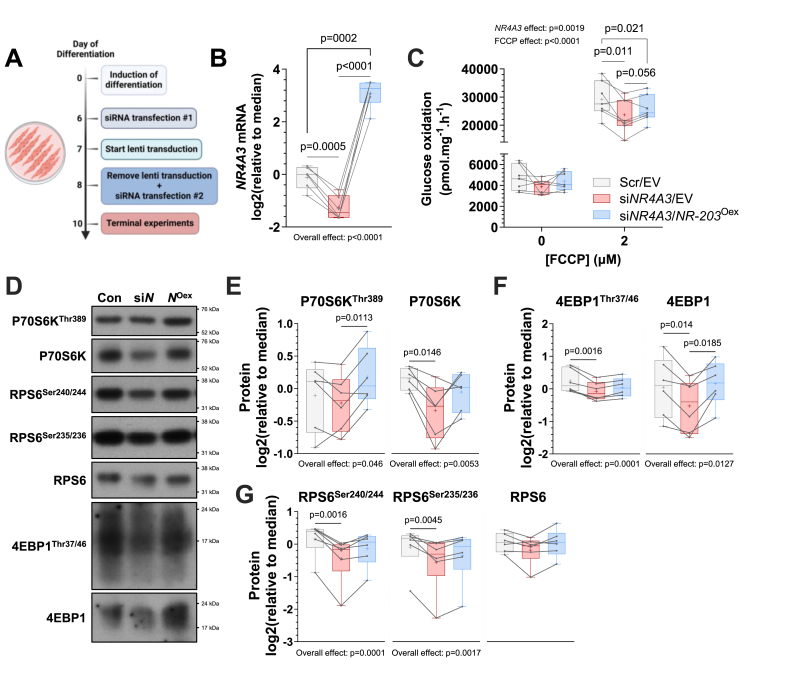
**Overexpression of the canonical NR4A3 isoform partially restores glucose oxidation after global *NR4A3* silencing in myotubes, concomitant with rescued mTORC1 signalling. A.** Schematic representation of the *NR4A3* ‘rescue’ experiment. **B.***NR4A3* mRNA expression measured by RT-qPCR. *n* = 6, 1-way ANOVA with Tukey correction. **C.** Rates of radiolabelled glucose oxidation under basal and (2 μM) FCCP-stimulated conditions over 4 h. *n* = 7, 2-way ANOVA (*NR4A3* x FCCP) with Fisher’s LSD post-test. **D.** Immunoblot of proteins in the P70S6K-RPS6 and 4EBP1 branches of the mTORC1 pathway from an indicative donor and **E-G.** quantification of each depicted signalling event. *n* = 6 and either 1-way ANOVA with Tukey correction or Friedman test with Dunn’s correction was performed. Overall statistical model effects are stated in relevant figures.

Considering our finding that canonical *NR4A3* gain-of-function augmented translation, we next tested whether this was sufficient to attenuate pharmacologically evoked reductions in myotube size. Dexamethasone is a synthetic glucocorticoid that, like disuse muscle loss in humans [3,8,15], rapidly promotes atrophy *in vitro* [51] and *in vivo* primarily through attenuation of protein synthesis [52]. Notably, after 48 h of dexamethasone exposure, myotubes transduced with *NR4A3-203* were resistant to glucocorticoid-induced atrophic effects, resulting in larger myotube areas compared to empty vector (EV) control (Figure 5E, F). Moreover, reconstitution of NR4A3-203, through overexpression of the canonical *NR4A3* transcript in the presence of siRNA targeting all *NR4A3* variants (Figure 6A–B), partially restored glucose oxidation (Figure 6C) concomitant with recovery of signalling downstream of mTORC1 (Figure 6D): ribosomal protein S6 kinase (P70S6K), RPS6, and 4EBP1 (Figure 6E–G). Despite these effects, AMPKα^Thr172^ phosphorylation remained elevated after restoration of NR4A3-203 (Supplementary Fig. 6A), which likely contributed to sustained upregulation of fatty acid oxidation rates (Supplementary Fig. 6B).

Overall, these results posit the NR4A3-203 isoform as a major contributor towards the negative metabolic outcomes associated with NR4A3-dependent suppression of protein synthesis and further imply sustained NR4A3 expression could circumvent decrements in skeletal muscle mass during periods of enforced inactivity.

### 3.5 *NR4A3* silencing alters primary human myotube size and structure

**Figure 7:**
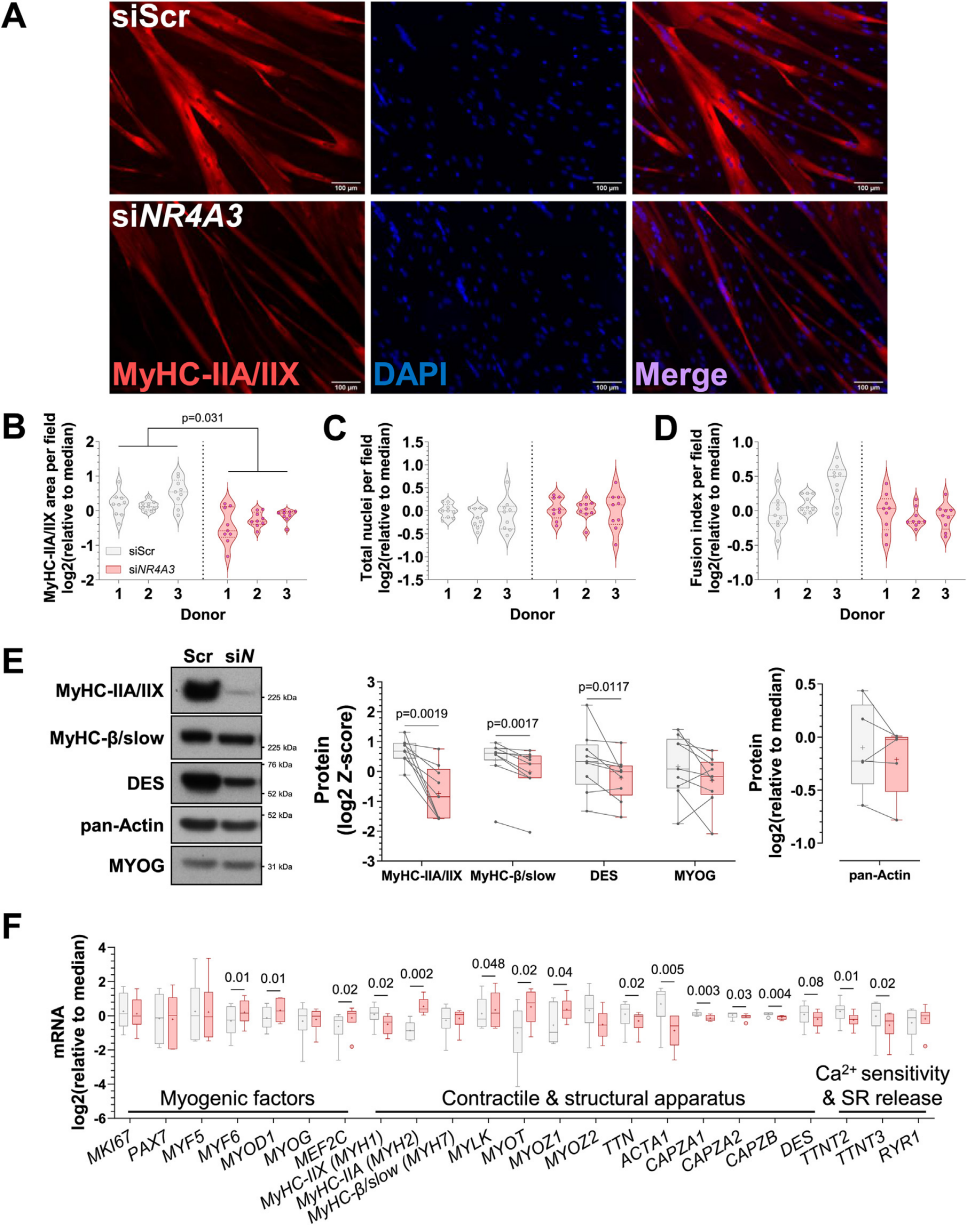
***NR4A3* silencing reduces myotube size and alters the expression of muscle contractile and structural apparatus.** Primary skeletal muscle cells were exposed to a control scramble sequence (siScr) or a silencing RNA targeting *NR4A3* (si*NR4A3*). **A.** Immunocytochemistry of fast myosin heavy chain isoforms (MYH1/2, red) and nuclei (DAPI, blue) from a representative donor. Scale bar = 100 μm. Quantification of immunocytochemistry for **B.** MyHC-IIA and IIX area, **C.** total nuclei, and **D.** fusion index per field of view. Results are violin plots with median and interquartile range. Circles represent measurements from different fields of view across three technical replicates per donor. *n* = 3, nested paired t-test. **E.** Representative immunoblot and quantification of proteins involved in contraction, structure, and myogenic transcription. Results for MyHC-IIA/IIX, MyHC-β/slow, DES, and MYOG are the average Z-score of log2-transformed values from basal conditions across experiments (*n* = 9, paired t-test). Results for pan-Actin are *n* = 5, 2-way ANOVA (silencing x insulin + leucine) with only basal data shown. **F.** mRNA expression of genes involved in myogenesis, muscle structure, and contraction measured by RT-qPCR. Results are box-and-whisker plots with Tukey distribution and crosses indicating mean values. *n* = 6, multiple paired t-tests or Wilcoxon signed-rank tests with FDR correction. ‘SR’ = sarcoplasmic reticulum. (For interpretation of the references to colour in this figure legend, the reader is referred to the Web version of this article.)

*NR4A3* is decreased during inactivity associated with muscle disuse (Figure 1A, B). We therefore examined how changes in translation elicited by *NR4A3* silencing impact on myotube size and excitation-contraction coupling machinery. Immunostaining for fast myosin heavy chain isoforms (MyHC-IIA and MyHC-IIX, encoded by *MYH2* and *MYH1*, respectively) revealed a striking decrease in myotube size (Figure 7A). This difference was driven by a reduction in myotube area (Figure 7B) without changes in the number of nuclei (Figure 7C) or the ability of myoblasts to fuse into myotubes (Figure 7D). NR4A3 depletion also reduced the protein abundance of fast and slow (MyHC-β, encoded by *MYH7*) myosin heavy chain isoforms, as well as the sarcolemma, Z-disc, and nuclear membrane integrating protein desmin (DES) (Figure 7E; Supplementary Fig. 7). These observations were consistent with *in vitro* [18] and *in vivo* [19,23,48] mouse studies of *Nr4a3* and further compounded by the altered expression of transcripts integral for skeletal muscle contraction and structure. This included downregulation of *MYH1*, titin (*TTN*), capping actin proteins of muscle Z-line (*CAPZ* family), and troponin isoforms *TTNT2* and *TTNT3* (Figure 7F).

Altogether, the protein and mRNA levels of major structural and contractile proteins of skeletal muscle cells were disrupted by *NR4A3* silencing. This associated with diminished myotube size and implies that NR4A3 is integral to skeletal muscle function.

## 4 Conclusions

*NR4A3* is markedly induced following a single exercise bout [30], but not after habitual exercise training [17]. Similarly, *NR4A3* is transiently downregulated during reduced physical activity and quickly upregulated upon reloading, but unchanged after prolonged immobilisation (>2 weeks). The onset of muscle fibre atrophy occurs rapidly with disuse [3,8] and is most aggressive across the initial two weeks of unloading [53], which coincides with the lowest levels of *NR4A3* mRNA. This expression pattern suggests an important role for NR4A3 in regulating skeletal muscle function during the acute transition phases associated with intense tissue remodelling. The first two weeks of unloading correspond to lower postprandial [14] and total daily [13] rates of myofibrillar protein synthesis. Here, we provide evidence that NR4A3 exerts its effects by directly influencing translation in skeletal muscle. Furthermore, NR4A3 downregulation in myotubes recapitulated adverse events observed in human [3,8,[13], [14], [15]] and rodent [34] skeletal muscle tissue following inactivity. This included impaired glucose metabolism, reduced protein synthesis, altered expression of the contractile apparatus, and diminished myotube size. Conversely, overexpression of the canonical NR4A3 isoform (*NR4A3-203*) enhanced translation and total cellular protein concentration, and conferred protection against glucocorticoid-induced reductions in myotube size. Thus, our results shed light on the involvement of NR4A3 in the control of skeletal muscle protein synthesis and metabolism during disuse atrophy.

Several lines of evidence link NR4A3 to metabolic responses in skeletal muscle. Rats bred for aerobic fitness have skeletal muscle enrichment of NR4A3, associated with higher mitochondrial content and increased running capacity [54]. In transgenic mice, tissue-specific *Nr4a3* overexpression promotes skeletal muscle remodelling towards an oxidative phenotype [19], suggesting that NR4A3 plays a causal role in metabolic reprogramming of skeletal muscle. These observations from *in vivo* studies substantiate results from *in vitro* experiments in mouse C2C12 and primary human skeletal myotubes, demonstrating that depletion of NR4A3 changes gene expression profiles towards anaerobic programmes [33] and impairs aspects of mitochondrial function [17,18,33]. Such effects may account for the lower ATP concentrations in C2C12 myotubes after *Nr4a3* silencing [33]. Our data add mechanistic evidence that NR4A3 downregulation profoundly impacts substrate utilisation, shifting metabolism towards increased lactate production and fatty acid oxidation, while decreasing glucose oxidation. Additionally, we corroborate findings in C2C12 [18] that mTORC1 is a signalling node inhibited by NR4A3 depletion. Activation of AMPK led to the phosphorylation of TSC2 and RPTOR at sites that blunt mTORC1 transduction [16,38,39]. However, we also observed decreased mTOR and RPS6 protein abundance; a phenomenon unlikely triggered exclusively by the activation of AMPK. Notably, rates of translation and ribosomal biogenesis were attenuated in response to *NR4A3* RNA interference, implying that reductions in NR4A3 impair cellular metabolism by obstructing cell-wide anabolic pathways. In agreement, rescuing protein synthetic signalling via reconstitution of NR4A3-203 restored glucose oxidation in *NR4A3*-silenced myotubes.

In aged mice, viral-mediated overexpression of *Nr4a3* improved muscle fatigue resistance and mitochondrial respiration [23]. Our study expands such observations by connecting lower levels of NR4A3 with inactivity paradigms and indicates that attenuation of NR4A3 has adverse effects on protein synthesis and metabolic responses in human skeletal muscle. While our analysis of NR4A3 expression presented herein focused on mRNA abundance, we also performed commensurate immunoblotting, confirming silencing and overexpression of NR4A3 protein in our respective models. In doing so, we detected differential patterns of NR4A3 molecular weight distribution across experimental conditions (Supplementary Fig. 8A, 8B). At present, little is known regarding the regulation of NR4A3 protein in human skeletal muscle or the stability and functional significance of individual NR4A3 isoforms. Thus, the variability of immunoblot profiles could stem from a range of biological processes, including alternative splicing [31,49], mRNA editing [55], or post-translational modifications, such as SUMOylation [56], ubiquitination [57], methylation, and phosphorylation [58]. Indeed, there are several phospho-sites on NR4A3 of unresolved function [58] highlighting the need for further studies to elucidate isoform-specific roles and better define NR4A3’s complex involvement in the metabolic rewiring of muscle to changes in physical activity.

In summary, we demonstrate robust, reproducible molecular and phenotypic effects of NR4A3 modulation on skeletal muscle metabolism. These results posit NR4A3 as a key molecular transducer, downregulation of which contributes to the deleterious health consequences associated with sedentary lifestyles. Muscle mass and strength are important predictors of mortality in intensive care patients [59] and such individuals typically experience 1.5–2% atrophy of the quadriceps muscles, per day, during the first week of admission alone [60]. Furthermore, although glucagon-like peptide-1 (GLP-1) receptor agonists provide a potent pharmaceutical intervention combatting obesity, the pronounced reductions in fat mass come at the expense of considerable losses in lean body mass [61]. Consequently, fully resolving and addressing NR4A3 regulation could have far-reaching implications for global health and constitutes an important target to mitigate the adverse effects of inactivity, sarcopenia, and metabolic disease.

## CRediT authorship contribution statement

**Jonathon A.B. Smith:** Writing – review & editing, Writing – original draft, Visualization, Methodology, Investigation, Formal analysis, Data curation, Conceptualization. **Brendan M. Gabriel:** Writing – review & editing, Supervision, Conceptualization. **Aidan J. Brady:** Methodology, Formal analysis. **Ahmed M. Abdelmoez:** Methodology. **Mladen Savikj:** Methodology. **Shane C. Wright:** Writing – review & editing, Methodology. **Stefania Koutsilieri:** Writing – review & editing, Methodology. **Romain Barrès:** Writing – review & editing, Methodology. **Volker M. Lauschke:** Writing – review & editing, Methodology. **Anna Krook:** Writing – review & editing, Supervision, Funding acquisition, Conceptualization. **Juleen R. Zierath:** Writing – review & editing, Supervision, Funding acquisition, Conceptualization. **Nicolas J. Pillon:** Writing – review & editing, Writing – original draft, Visualization, Supervision, Methodology, Investigation, Funding acquisition, Formal analysis, Data curation, Conceptualization.

## Declaration of competing interest

Volker M. Lauschke reports a relationship with HepaPredict AB that includes: board membership and equity or stocks. Volker M. Lauschke reports a relationship with Shanghai Hepo Biotechnology Ltd that includes: board membership and equity or stocks. Other authors declare that they have no competing financial interests or personal relationships that could have appeared to influence the work reported in this paper.

## Data Availability

Data will be made available on request.

## Footnotes

Appendix A Supplementary data to this article can be found online at https://doi.org/10.1016/j.molmet.2025.102200.
