# Supplementary material for: Inactivity-induced NR4A3 downregulation in human skeletal muscle affects glucose metabolism and translation: Insights from in vitro analysis
Source: Mol Metab. 2025 Jul 1;99:102200. doi: 10.1016/j.molmet.2025.102200 (PMC12275858; doi:10.1016/j.molmet.2025.102200)
Supplement: Multimedia component 1 [file mmc1.docx]

**Supplementary Tables**

**Supplementary Table 1. Assays used in Taqman RT-qPCR**

| Gene | Vendor | Assay ID |
| --- | --- | --- |
| *ACCa* (*ACACA*) | Thermo Fisher Scientific | Hs01046047_m1 |
| *ACTA1* | Thermo Fisher Scientific | Hs00559403_m1 |
| *ACCb* (*ACACB*) | Thermo Fisher Scientific | Hs01565956_m1 |
| *AKT2* | Thermo Fisher Scientific | Hs01086102_m1 |
| *AMPKα_1_* (*PRKAA1*) | Thermo Fisher Scientific | Hs00178893_m1 |
| *AMPKα_2_* (*PRKAA2*) | Thermo Fisher Scientific | Hs00178903_m1 |
| *AR* (*androgen receptor*) | Thermo Fisher Scientific | Hs00907243_m1 |
| *ATF3* | Thermo Fisher Scientific | Hs00231069_m1 |
| *ATF4* | Thermo Fisher Scientific | Hs00909569_g1 |
| *B2M* | Thermo Fisher Scientific | Hs00187842_m1 |
| *CAPZA1* | Thermo Fisher Scientific | Hs04187789_g1 |
| *CAPZA2* | Thermo Fisher Scientific | Hs00255135_m1 |
| *CAPZB* | Thermo Fisher Scientific | Hs01120796_m1 |
| *CD36* | Thermo Fisher Scientific | Hs00354519_m1 |
| *CHOP* (*DDIT3*) | Thermo Fisher Scientific | Hs00358796_g1 |
| *DES* | Thermo Fisher Scientific | Hs00157258_m1 |
| *EIF4E* | Thermo Fisher Scientific | Hs00854166_g1 |
| *FABP3* | Thermo Fisher Scientific | Hs00997360_m1 |
| *FASN* | Thermo Fisher Scientific | Hs01005622_m1 |
| *FATBP1* (*SLC27A1*) | Thermo Fisher Scientific | Hs01587917_m1 |
| *FATP4* (*SLC27A4*) | Thermo Fisher Scientific | Hs00192700_m1 |
| *GYS1* | Thermo Fisher Scientific | Hs00157863_m1 |
| *GLUT1* (*SLC2A1*) | Thermo Fisher Scientific | Hs00892681_m1 |
| *GSK3B* | Thermo Fisher Scientific | Hs01047719_m1 |
| *HPRT1* | Thermo Fisher Scientific | Hs02800695_m1 |
| *IGF1R* | Thermo Fisher Scientific | Hs00609566_m1 |
| *IL6* | Thermo Fisher Scientific | Hs00174131_m1 |
| *INSR* | Thermo Fisher Scientific | Hs00961554_m1 |
| *LDHB* | Thermo Fisher Scientific | Hs00929956_m1 |
| *MCT1* (*SLC16A1*) | Thermo Fisher Scientific | Hs00161826_m1 |
| *MCT4* (*SLC16A3*) | Thermo Fisher Scientific | Hs00358829_m1 |
| *MEF2C* | Thermo Fisher Scientific | Hs00231149_m1 |
| *MKI67* | Thermo Fisher Scientific | Hs04260396_g1 |
| *MSTN* | Thermo Fisher Scientific | Hs00976237_m1 |
| *MuRF1* (*TRIM63*) | Thermo Fisher Scientific | Hs00822397_m1 |
| *MyHC-IIX* (*MYH1*) | Thermo Fisher Scientific | Hs00428600_m1 |
| *MyHC-IIA* (*MYH2*) | Thermo Fisher Scientific | Hs00430042_m1 |
| *MyHC-β/slow* (*MYH7*) | Thermo Fisher Scientific | Hs01110632_m1 |
| *MYF5* | Thermo Fisher Scientific | Hs00929416_g1 |
| *MYF6* | Thermo Fisher Scientific | Hs00231165_m1 |
| *MYLK* | Thermo Fisher Scientific | Hs00364926_m1 |
| *MYOD1* | Thermo Fisher Scientific | Hs00159528_m1 |
| *MYOG* | Thermo Fisher Scientific | Hs01072232_m1 |
| *MYOT* | Thermo Fisher Scientific | Hs00199016_m1 |
| *MYOZ1* | Thermo Fisher Scientific | Hs00222007_m1 |
| *MYOZ2* | Thermo Fisher Scientific | Hs00213216_m1 |
| *NR4A1* | Thermo Fisher Scientific | Hs00374226_m1 |
| *NR4A2* | Thermo Fisher Scientific | Hs01117527_g1 |
| *NR4A3* | Thermo Fisher Scientific | Hs00545009_g1 |
| *NRF1* | Thermo Fisher Scientific | Hs00602161_m1 |
| *NRF2* (*NFE2L2*) | Thermo Fisher Scientific | Hs00975961_g1 |
| *PAX7* | Thermo Fisher Scientific | Hs00242962_m1 |
| *PDK4* | Thermo Fisher Scientific | Hs01037712_m1 |
| *PKM2* | Thermo Fisher Scientific | Hs00762869_s1 |
| *PPARα* (*PPARA*) | Thermo Fisher Scientific | Hs00231882_m1 |
| *PPARδ* (*PPARD*) | Thermo Fisher Scientific | Hs00602622_m1 |
| *P70S6K* (*RPS6KB1*) | Thermo Fisher Scientific | Hs00356369_m1 |
| *PSMA1* | Thermo Fisher Scientific | Hs00267631_m1 |
| *PSMA6* | Thermo Fisher Scientific | Hs00930456_m1 |
| *PSMB2* | Thermo Fisher Scientific | Hs01002946_m1 |
| *RPL32* | Thermo Fisher Scientific | Hs00851655_g1 |
| *RPS6* | Thermo Fisher Scientific | Hs01058685_g1 |
| *RYR1* | Thermo Fisher Scientific | Hs00166991_m1 |
| *Eukaryotic 18S rRNA* | Applied Biosystems | Hs99999901_s1 |
| *SESN2* | Thermo Fisher Scientific | Hs00900115_m1 |
| *TNNT2* | Thermo Fisher Scientific | Hs00165960_m1 |
| *TNNT3* | Thermo Fisher Scientific | Hs00952980_m1 |
| *TTN* | Thermo Fisher Scientific | Hs00399225_m1 |

**Supplementary Table 2. Oligonucleotide sequences used in SYBR Green RT-qPCR**

| Gene | Fwd sequence | Rev sequence |
| --- | --- | --- |
| *CPT1B* | CATGTATCGCCGTAAACTGGAC | TGGTAGGAGCACATAGGCACT |
| *FOXO3* | CGGACAAACGGCTCACTCT | GGACCCGCATGAATCGACTAT |
| *GSK3A* | GAGGTTCAAGAACCGAGAGC | AGTGGATGTAGGCCAAGCTG |
| *GUSB* | GCAGATGTGTGACCGCTATG | TGAGCGATCACCATCTTCAAG |
| *HK2* | CAAGAAGCTCCCACTGGGTT | AAAGTCCCCTCTCCTCTGGA |
| *LARS1* | GAATGGGTTTGAAGGTAGACTGG | GCTGTCCATCTTTCGGAGAGT |
| *LAT1* (*SLC7A5*) | CCGTGAACTGCTACAGCGT | CTTCCCGATCTGGACGAAGC |
| *LDHA* | ACGTCAGCAAGAGGGAGAAA | CGCTTCCAATAACACGGTTT |
| *MYC* | GCTGCTTAGACGCTGGATTT | CACCGAGTCGTAGTCGAGGT |
| *NR4A3* | GCCCAGTAGACAAGAGACGT | GAGAGGGCTGAGAAGGTTCC |
| *PHGDH* | CTGCGGAAAGTGCTCATCAGT | TGGCAGAGCGAACAATAAGGC |
| *RPL3* | AAGTCGACAGGCCGGGATC | CTTCTTCTTAGATTTATGCCAA |
| *RPL11* | TCCACTGCACAGTTCGAGGG | AAACCTGGCCTACCCAGCAC |
| *RPLP0* | AGCCCAGAACACTGGTCTC | ACTCAGGATTTCAATGGTGCC |
| *SLC1A5* | TCATGTGGTACGCCCCTGT | GCGGGCAAAGAGTAAACCCA |
| *45S pre-rRNA* (*5' ETS*) | GAACGGTGGTGTGTCGTT | GCGTCTCGTCTCGTCTCACT |
| *45S pre-rRNA* (*28S 5' ITS*) | TCCGAGACGCGACCTCAG | TCGCCGTTACTGAGGGAATC |
| *5S rRNA* | GGCCATACCACCCTGAACGC | CAGCACCCGGTATTCCCAGG |
| *5.8S rRNA* | ACTCGGCTCGTGCGTC | GCGACGCTCAGACAGG |
| *28S rRNA* | GTGACGCGCATGAATGGA | TGTGGTTTCGCTGGATAGTAGGT |
| *TBP* | AGTTCTGGGATTGTACCGCA | TATATTCGGCGTTTCGGGCA |
| *TFEB* | GCGGCAGAAGAAAGACAATC | CTGCATCCTCCGGATGTAAT |

5’ ETS = 5-prime external transcribed spacer, 5’ ITS = 5-prime internal transcribed spacer.

**Supplementary Table 3. Antibodies used for immunoblotting**

| Protein | Vendor | Catalogue # | Research resource ID # | Dilution |
| --- | --- | --- | --- | --- |
| ACC^Ser79^ | Cell Signaling | 3661 | AB_330337 | 1:1000 |
| ACC | Cell Signaling | 3676 | AB_2219397 | 1:1000 |
| AKT^Ser473^ | Cell Signaling | 9271 | AB_329825 | 1:1000 |
| AKT^Thr308^ | Cell Signaling | 4056 | AB_331163 | 1:1000 |
| AKT | Cell Signaling | 9272 | AB_329827 | 1:1000 |
| AMPKα^Thr172^ | Cell Signaling | 2531 | AB_330330 | 1:1000 |
| AMPKα | Cell Signaling | 2532 | AB_330331 | 1:1000 |
| AS160^Thr642^ | Cell Signaling | 8881 | AB_2651042 | 1:1000 |
| AS160 | Abcam | ab24469 | AB_778263 | 1:1000 |
| ATGL | Abcam | ab109251 | AB_10864772 | 1:1000 |
| β-tubulin | Cell Signaling | 2128 | AB_823664 | 1:1000 |
| Calpain 1 | Abcam | ab28258 | AB_725819 | 1:1000 |
| Caspase 3 | Cell Signaling | 9662 | AB_331439 | 1:1000 |
| CPT1B | Abgent | AP2532B | AB_2292207 | 1:1000 |
| DES | Abcam | Ab15200 | AB_301744 | 1:1000 |
| 4EBP1^Thr37/46^ | Cell Signaling | 9459 | AB_330985 | 1:1000 |
| 4EBP1 | Cell Signaling | 9452 | AB_331692 | 1:1000 |
| ERK1/2^Thr202/Tyr204^ | Cell Signaling | 9101 | AB_331646 | 1:1000 |
| ERK1/2 | Cell Signaling | 4695 | AB_390779 | 1:1000 |
| FOXO1^Ser256^ | Cell Signaling | 9461 | AB_329831 | 1:1000 |
| FOXO1 | Cell Signaling | 2880 | AB_2106495 | 1:1000 |
| FOXO3a^Ser253^ | Cell Signaling | 13129 | AB_2687495 | 1:1000 |
| FOXO3a | Abcam | Ab47409 | AB_869815 | 1:1000 |
| GS^Ser641^ | Cell Signaling | 3891 | AB_2116390 | 1:1000 |
| GS | Cell Signaling | 3893 | AB_2279563 | 1:1000 |
| GSK3α/β^Ser21/9^ | Cell Signaling | 9331 | AB_329830 | 1:1000 |
| GSK3α/β | Cell Signaling | 5676 | AB_10547140 | 1:1000 |
| H3 | Cell Signaling | 9715 | AB_331563 | 1:1000 |
| HK2 | Cell Signaling | 2867 | AB_2232946 | 1:1000 |
| HSL^Ser660^ | Cell Signalling | 45804 | AB_2893315 | 1:1000 |
| IκBα | Cell Signalling | 9242 | AB_331623 | 1:1000 |
| LC3 | Sigma-Aldrich | L8918 | AB_1079382 | 1:1000 |
| LDH | Abcam | ab52488 | AB_2134961 | 1:1000 |
| LIPIN-1 (N-TERM) | Chemicon | AB3585 | AB_11210988 | 1:1000 |
| MaFbx | Santa Cruz | 166806 | AB_2246982 | 1:1000 |
| mTOR^Ser2448^ | Cell Signaling | 2971 | AB_330970 | 1:1000 |
| mTOR | Cell Signaling | 2983 | AB_2105622 | 1:1000 |
| MyHC-IIA/IIX (MYH1/2) | Santa Cruz | 53088 | AB_784722 | 1:1000 |
| MyHC-β/slow (MYH7) | Santa Cruz | 53089 | AB_2147281 | 1:1000 |
| MYOG | Santa Cruz | 12732 | AB_627980 | 1:1000 |
| MuRF1 | Santa Cruz | 134397 | AB_2208860 | 1:1000 |
| NR4A3 | Novus Biologicals | NBP2-46246 | AB_3310257 | 1:1000 |
| pan-20Sα | Abcam | Ab22674 | AB_2171376 | 1:1000 |
| pan-Actin | Cell Signaling | 8456 | AB_10998774 | 1:1000 |
| PDHA1^Ser293^ | Cell Signaling | 37115 | AB_2923272 | 1:1000 |
| Puromycin (clone 12D10) | Sigma-Aldrich | MABE343 | AB_2566826 | 1:1000 |
| PYGM | Abcam | 88078 | AB_2042744 | 1:1000 |
| p62 (SQSTM1) | Sigma-Aldrich | P0067 | AB_1841064 | 1:1000 |
| P70S6K^Thr389^ | Cell Signaling | 9205 | AB_330944 | 1:1000 |
| P70S6K | Cell Signaling | 2708 | AB_390722 | 1:1000 |
| P90RSK^Thr573^ | Cell Signaling | 9346 | AB_330795 | 1:1000 |
| RPS6^Ser235/236^ | Cell Signaling | 2211 | AB_331679 | 1:1000 |
| RPS6^Ser240/244^ | Cell Signaling | 5364 | AB_10694233 | 1:1000 |
| RPS6 | Cell Signaling | 2217 | AB_331355 | 1:1000 |
| RPTOR^Ser792^ | Cell Signaling | 2083 | AB_2249475 | 1:1000 |
| RPTOR | Cell Signaling | 2280 | AB_561245 | 1:1000 |
| RSK2 | Cell Signaling | 9340 | AB_2181471 | 1:1000 |
| TBC1D1^Ser237^ | Millipore | 07-2268 | AB_10807809 | 1:1000 |
| TBC1D1 | Cell Signaling | 4629 | AB_1904162 | 1:1000 |
| TSC2^Ser1387^ | Cell Signaling | 5584 | AB_10698883 | 1:1000 |
| TSC2^Thr1462^ | Cell Signaling | 3617 | AB_490956 | 1:1000 |
| TSC2 | Cell Signaling | 4308 | AB_10547134 | 1:1000 |
| Ubiquitin (FK2) | Millipore | 04-263 | AB_612093 | 1:1000 |
| ULK1^Ser317^ | Cell Signaling | 12753 | AB_2687883 | 1:1000 |

**Supplementary Figures**

**Supplementary Figure 1. *NR4A3*-silenced myotubes retain responsiveness to insulin stimulation.** Primary skeletal muscle cells were exposed to a control scramble sequence (siScr) or a silencing RNA targeting *NR4A3* (si*NR4A3*). **A.** Representative immunoblot and **B-G.** quantification of proteins and phosphorylation events in the canonical insulin signalling cascade. n = 6, 2-way ANOVA (silencing x insulin) with Šidák correction. **H.** Rates of radiolabelled 2-Deoxy-D-glucose uptake (n = 7) and **I.** glycogen synthesis (n = 6) under basal and insulin-stimulated conditions. 2-way ANOVA (silencing x insulin) with Šidák correction. Overall statistical model effects are stated in figures. When abbreviated, si*N* = main silencing effect and si*N* x Ins = silencing x insulin interaction effect. **J.** Representative immunoblot and quantification of total glycogen phosphorylase, muscle associated (PYGM) and **K.** lactate dehydrogenase (LDH). n = 5, 2-way ANOVA (silencing x insulin + leucine). Only basal data are shown.

**Supplementary Figure 2. *NR4A3* silencing alters phosphorylation events and total protein abundance within the AMPK signalling cascade.** Primary skeletal muscle cells were exposed to a control scramble sequence (siScr) or a silencing RNA targeting *NR4A3* (si*NR4A3*). **A.** Immunoblot quantification of activating phosphorylation sites and total protein levels for AMPKα and the AMPK substrates **B.** ACC and **C.** TBC1D1. n = 5, 2-way ANOVA (silencing x insulin + leucine) with Šidák correction. Only basal data are shown and overall effects in the model are presented for multiple comparisons p>0.05 but si*NR4A3* main effects p<0.1.

**Supplementary Figure 3. *NR4A3* silencing attenuates mTORC1 signalling.** Primary skeletal muscle cells were exposed to a control scramble sequence (siScr) or a silencing RNA targeting *NR4A3* (si*NR4A3*). **A.** Immunoblot quantification of phosphorylated-to-total protein ratios for AMPK target sites on RPTOR (Ser792) and TSC2 (Ser1387). **B.** Immunoblot quantification of total protein abundance for RPTOR, and AKT-mTORC1 activating phosphorylation sites and total protein levels for **C**. mTOR, **D.** RPS6, **E.** 4EBP1 (with images from a representative donor), **F.** TSC2, and **G.** AKT. All immunoblot analyses are n = 5, 2-way ANOVA (silencing x insulin + leucine) with Šidák correction; except 4EBP1, which is n = 6, 2-way ANOVA (silencing x insulin) with Šidák correction, and AKT^Ser473^ and AKT^Thr308^ phosphorylation, which are n = 5, paired t-tests. Overall statistical model effects are stated in figures. si*N* = main silencing effect, I+L = main insulin + leucine effect, si*N* x I+L = interaction effect.

**Supplementary Figure 4. *NR4A3* silencing minimally impacts pathways controlling protein degradation.** Primary skeletal muscle cells were exposed to a control scramble sequence (siScr) or a silencing RNA targeting *NR4A3* (si*NR4A3*). **A.** mRNA expression of genes related to the negative regulation of muscle mass, endoplasmic reticulum (ER) stress, oxidative stress, and inflammation measured by RT-qPCR. Results are box-and-whisker plots with Tukey distribution and crosses indicating mean values. n = 6, paired t-tests with FDR correction. **B.** Immunoblot of proteins involved in inflammation, ubiquitin-proteasomal, and autophagy-lysosomal degradation pathways from a representative donor. **C-G.** Immunoblot quantification of **C.** NF-κB inhibitor IκBα, basal and insulin-mediated inhibitory phosphorylation of transcription factors **D.** FOXO3a, **E.** FOXO1, and E3 ubiquitin ligases **F.** MuRF1 and **G.** MAFbx**.** **H.** Representative immunoblot and quantification of cellular protein ubiquitination. **I-K.** Immunoblot quantification of **I.** Calpain-1 and **J.** Caspase 3 proteases, and **K.** alpha subunits of the 20S core particle proteasome (pan-20Sα). **L-N.** Immunoblot quantification of autophagy markers **L.** ULK1 phosphorylation at Ser317, and total **M.** p62 and **N.** LC3-I/LC3-II abundance. All immunoblot analyses are n = 5, 2-way ANOVA (silencing x insulin + leucine) with Šidák correction. Overall statistical model effects are stated in figures.

**Supplementary Figure 5. Overexpression of the canonical *NR4A3* isoform increases translation without altering glucose and fatty acid metabolism, RNA abundance, or mTORC1 signalling.** Primary skeletal muscle cells were transduced with an empty vector control plasmid (EV) or a plasmid containing variant *NR4A3-203* (*NR-203*^Oex^). **A.** Basal and stimulated rates of radiolabelled 2-Deoxy-D-glucose uptake, **B.** glycogen synthesis, **C.** glucose oxidation, and **D.** ^14^C palmitic acid oxidation. n = 6, 2-way ANOVA (overexpression x stimulation). Overall statistical model effects are stated in figures. **E.** Total RNA concentration (ng.μL^-1^). n = 6, paired t-test. **F.** Immunoblot of proteins in the mTORC1 and ERK signalling cascades from a representative donor. Veh = vehicle, Rapa = rapamycin, and Cyclo = cycloheximide treatments, respectively. Samples are those presented in main **Figure 5B**. No difference in phosphorylation or total protein levels were detected with *NR-203*^Oex^. n = 5, 3-way ANOVA (overexpression x rapamycin x insulin + leucine). **G.** Volcano plot of all mRNA transcripts assessed after *NR-203*^Oex^ measured by RT-qPCR. Genes were the same as analysed in *NR4A3*-silenced conditions. n = 6, multiple paired t-tests with FDR correction.

**Supplementary Figure 6. Reconstitution of the canonical *NR4A3* isoform after global NR4A3-depletion does not ameliorate AMPKα phosphorylation or fatty acid oxidation.** Primary skeletal muscle cells were depleted of all *NR4A3* transcripts using siRNA and then transduced with an empty vector control plasmid or a plasmid containing variant *NR4A3-203* as described in main **Figure 6A**. **A.** Representative immunoblot and quantification of total and phosphorylated AMPKα. n = 6, 1-way ANOVA with Tukey correction. **B.** Rates of radiolabelled palmitic acid oxidation under basal and (2 µM) FCCP-stimulated conditions over 4 h. n = 6, 2-way ANOVA (*NR4A3* x FCCP) with Fisher’s LSD post-test. Overall statistical model effects are stated in figures.

**Supplementary Figure 7. The effect of *NR4A3* silencing on contractile proteins is specific to siRNA targeting *NR4A3* transcripts.** Primary skeletal muscle cells were exposed to a control scramble sequence (Scr) or a silencing RNA targeting *NR4A3* (si*N*). **A.** Representative immunoblot of myosin heavy chain isoforms IIA (MyHC-IIA, encoded by *MYH2*) and IIX (MyHC-IIX, encoded by *MYH1*) from three independent donors. ‘NT’ = non-transfected condition.

**Supplementary Figure 8. Effects of NR4A3 silencing and overexpression are reflected at the protein level; however, NR4A3 protein distribution varies across experimental conditions.** **A.** Primary skeletal muscle cells were exposed to a control scramble sequence (siScr) or a silencing RNA targeting *NR4A3* (si*NR4A3*). Representative immunoblot and quantification of NR4A3 protein abundance. Results are the average Z-score of log2-transformed values from basal conditions across experiments (n = 9, paired t-test). **B.** Primary skeletal myotubes were transduced with an empty vector control plasmid (EV) or a plasmid containing variant *NR4A3-203* (*NR-203*^Oex^). Indicative immunoblot and determination of NR4A3 protein levels upon overexpression. n = 5, 2-way ANOVA (silencing x insulin + leucine) with Šidák correction. Note, to remain consistent between models of *NR4A3* manipulation, analyses represent quantification of higher molecular weight NR4A3, as depicted in A. However, similar results and significance were obtained when all 52-76 kDa bands were assessed.
